# Exosomal miRNA profiling from H5N1 avian influenza virus-infected chickens

**DOI:** 10.1186/s13567-021-00892-3

**Published:** 2021-03-03

**Authors:** Yeojin Hong, Anh Duc Truong, Jiae Lee, Thi Hao Vu, Sooyeon Lee, Ki-Duk Song, Hyun S. Lillehoj, Yeong Ho Hong

**Affiliations:** 1 Department of Animal Science and Technology, Chung-Ang University, Anseong, 17546 Republic of Korea; 2 Department of Biochemistry and Immunology, National Institute of Veterinary Research, 86 Truong Chinh, Dong Da, Hanoi, 100000 Vietnam; 3 Department of Animal Biotechnology, College of Agricultural and Life Sciences, Jeonbuk National University, Jeonju, 54896 Republic of Korea; 4 Animal Biosciences and Biotechnology Laboratory, Agricultural Research Services, United States Department of Agriculture, Beltsville, MD 20705 USA

**Keywords:** H5N1, HPAIV, Exosomes, Small RNA sequencing, miRNA

## Abstract

Exosomes are membrane vesicles containing proteins, lipids, DNA, mRNA, and micro RNA (miRNA). Exosomal miRNA from donor cells can regulate the gene expression of recipient cells. Here, Ri chickens were divided into resistant (*Mx*/A; *BF2*/*B21*) and susceptible (*Mx*/G; *BF2*/*B13*) trait by genotyping of *Mx* and *BF2* genes. Then, Ri chickens were infected with H5N1, a highly pathogenic avian influenza virus (HPAIV). Exosomes were purified from blood serum of resistant chickens for small RNA sequencing. Sequencing data were analysed using FastQCv0.11.7, Cutadapt 1.16, miRBase v21, non-coding RNA database, RNAcentral 10.0, and miRDeep2. Differentially expressed miRNAs were determined using statistical methods, including fold-change, exactTest using edgeR, and hierarchical clustering. Target genes were predicted using miRDB. Gene ontology analysis was performed using gProfiler. Twenty miRNAs showed significantly different expression patterns between resistant control and infected chickens. Nine miRNAs were up-regulated and 11 miRNAs were down-regulated in the infected chickens compared with that in the control chickens. In target gene analysis, various immune-related genes, such as cytokines, chemokines, and signalling molecules, were detected. In particular, mitogen-activated protein kinase (MAPK) pathway molecules were highly controlled by differentially expressed miRNAs. The result of qRT-PCR for miRNAs was identical with sequencing data and miRNA expression level was higher in resistant than susceptible chickens. This study will help to better understand the host immune response, particularly exosomal miRNA expression against HPAIV H5N1 and could help to determine biomarkers for disease resistance.

## Introduction

Exosomes are membrane vesicles, approximately 40–100 nm in diameter, and present in most biological fluids [1–3]. Exosomes are derived from multivesicular bodies (MVBs) to form intraluminal vesicles (ILVs), which are then released into the extracellular environment as exosomes after fusion with the plasma membrane [3]. Lipids and proteins are the main components of exosomes and various nucleic acids, such as mRNAs, microRNAs (miRNAs), and other non-coding RNAs (ncRNAs), and are also found in the exosomal lumen [1–4]. These exosomal RNAs can be delivered from donor cells to recipient cells, wherein they modulate various biological systems [5–7]. Therefore, exosomes are important in cell-to-cell communication.

miRNAs are small ncRNA molecules and are typically 22 nucleotides in length, which repress translation of the target mRNA by binding to the 3′-untranslated region and/or induce the decay of up to 30% of all expressed transcripts [8]. miRNAs are involved in diverse biological processes, including fat metabolism; cell death, proliferation, differentiation; and the functioning of the immune system [9]. Therefore, exosomal miRNAs from donor cells can regulate the gene expression of recipient cells. Furthermore, the composition of exosomal miRNAs is different between healthy and diseased individuals [10]. Thus, exosomal miRNAs indicate the state of disease and control the immune systems.

Avian influenza viruses (AIV) in *Influenzavirus A* genus, belonging to the family *Orthomyxoviridae*, cause severe outbreaks in the poultry industry. In particular, the H5N1 subtype is a highly pathogenic avian influenza virus (HPAIV) that originated from Asia [11]. H5N1 in poultry decreases egg production and causes rhinorrhoea, loss of appetite, soft-shelled or misshapen eggs, diarrhoea, and sudden death [12]. Thus, H5N1 outbreak causes significant economic damage to the poultry industry. Furthermore, H5N1 can be transmitted to humans and causes severe acute respiratory infection with a fatality rate of more than 50% [13]. Therefore, it is essential to elucidate the mechanisms of AIV pathogenesis in chickens to control the infection.

Exosomes have various roles in immune response by delivering exosomal contents to other cells such as membrane-bound receptors and miRNAs [14]. Therefore, we suggest that exosomes might have important roles, especially delivering miRNAs, in immune response against infection of H5N1 HPAIV. To date, however, there are no studies describing exosomal small RNA transcriptome analysis in AIV infection. In this study, Ri chickens, local yellow-feathered household chickens of Vietnam [15], were used as experimental animals. Further, by genotyping the *Mx* and *BF2* gene, Ri chickens resistant and susceptible to AIV were distinguished. The Mx protein, which is part a of the dynamin family of large GTPases, interferes with the replication of RNA viruses by inhibiting trafficking or the activity of viral polymerases [16–18]. The chicken major histocompatibility complex (MHC) consist of *BLB* (Class II) and two *BF* (Class I) [19, 20]. Especially, among haplotype of *BF2*, *B21* allele is associated with high antibody titer against infectious bursal disease virus [21, 22]. Moreover, the chickens with *B21* haplotype have high survival rate and with *B13* have high mortality rate against H5N1 AIV [23]. To identify the profiling of exosomal miRNA against AIV infection, we infected Ri chickens with the AIV subtype H5N1 and purified exosomes from the serum for small RNA sequencing and analysis.

## Materials and methods

### Experimental birds and genotyping

All specific-pathogen-free (SPF) Ri chickens [15], a native Vietnamese chicken breed, were infected with AIV and observed daily for signs of disease and mortality. All chicken experiments were performed in our collaborative laboratory at the Department of Biochemistry and Immunology in the National Institute of Veterinary Research, Vietnam. A total of 40 Ri chickens belonging to resistant and susceptible lines were used in our study (see Additional file 1).

Genotyping of *Mx* and *BF2* was performed by high resolution melt analysis for resistance and susceptibility selection [16–18, 22, 23]. In detail, the polymorphism of allele A/G of *Mx* at position 631 demonstrated allele A (resistant trait) and allele G (susceptible trait) in Ri chickens (see Additional file 2). Among *BF2* haplotype, Ri chickens that have *B21* haplotype were selected as a resistant trait and *B13* haplotype were selected as a susceptible trait. Finally, Ri chickens *Mx*(A)/*B21* were selected as a resistant trait and *Mx*(G)/*B13* were selected as a susceptible trait. For HPAIV challenge, a total of 20 4-week-old birds, 10 Ri chickens in each line, received intranasal inoculation of 200 µL of harvested allantois fluid of the infected eggs, containing 1 × 10^4^ 50% egg infectious dose (EID_50_) [24] of A/duck/Vietnam/QB1207/2012 (H5N1), according to the OIE guideline.

### Exosome extraction and characterization

Blood samples were collected from the wing vein of chickens after 1 and 3 days of infection (three chickens from each group). Exosomes were extracted from the serum using Total Exosome Isolation Reagent (Invitrogen, Carlsbad, CA, USA), according to the manufacturer’s protocol. Briefly, 5 mL of the blood sample was collected from infected and control chickens. The blood was incubated at room temperature (RT) for 2 h to allow clotting. Serum was isolated from the clotted blood and centrifuged at 2000 × *g* for 30 min at 4 °C to remove cells and debris. The supernatant was mixed with 0.2 volumes of the Total Exosome Isolation reagent and incubated at 4 °C for 30 min. After incubation, samples were centrifuged at 10 000 × *g* for 10 min at RT. The supernatant was discarded, and exosomes were contained in the pellet at the bottom of the tube. Exosomes were suspended with phosphate-buffered saline (PBS; pH 7.4) and stored at ≤  −20 °C.

For characterization of exosomes, the particle size was measured using a Nanoparticle Analyzer (HORIBA, SZ-100, Kyoto, Japan). Furthermore, a Western blot assay was performed using CD81 as a exosomal marker (#56039; Cell Signaling Technology, Danvers, MA, USA) according to previously described methods [25].

### Exosomal RNA extraction and small RNA sequencing

Small RNA sequencing was conducted using exosomes from resistant Ri chickens at day 3 post-infection. Exosomal RNA was extracted using the miRNeasy Serum/Plasma Kit (Qiagen, Hilden, Germany) according to the manufacturer’s protocol. Library construction and small RNA sequencing were only conducted for resistant Ri chickens with 1 control (Resistant day-3) and 2 infection (Resistant day-3) samples. The library was constructed using SMARTer smRNA-Seq Kit for Illumina (TAKARA Bio Inc., Otsu, Shiga, Japan) according to the manufacturer’s protocol. Next, small RNA sequencing was conducted by Macrogen (Seoul, Republic of Korea) using a HiSeq 2500 System (Illumina Inc., San Diego, CA, USA).

### Bioinformatic analysis of sequencing data

The raw sequence reads were filtered based on quality using FastQC v0.11.7 [26]. Adapter sequences were trimmed off the raw sequence reads using Cutadapt 1.16 [27]. Both the trimmed and non-adapter reads were used as processed reads to analyze long targets (≥ 50 bp). Unique clustered reads were sequentially aligned to the reference genome using miRBase v21 [28], and the non-coding RNA database, RNAcentral 10.0 [29], to classify known miRNAs and other RNA types, such as tRNA, small nuclear RNA (snRNA), and small nucleolar RNA (snoRNA). Novel miRNA prediction was performed by miRDeep2 [30]. The read counts for each miRNA were extracted from mapped miRNAs to report the abundance of each miRNA. Differentially expressed miRNAs were determined by comparing each miRNA across conditions using statistical methods, such as fold-change, exactTest using edgeR (Empirical Analysis of Digital Gene Expression Data in R), and hierarchical clustering. Target genes of differentially expressed miRNAs were predicted using miRDB [31] and target genes with a target score over 80 were selected. Next, GO functional enrichment analysis of target genes was performed using gProfiler [32]. The miRNA-mRNA network were constructed using miRNet [33].

### miRNA primer design

Quantitative real-time PCR (qRT-PCR) for miRNAs only required a forward primer to be designed for the individual miRNA. The reverse primer was a universal primer provided with the miScript SYBR Green PCR Kit (Qiagen). All known chicken miRNA sequences were obtained from miRBase [34]. Oligonucleotide primers for these miRNAs were designed using full-length mature miRNA sequences. Primers were synthesized by Genotech (Daejeon, South Korea) (see Additional file 3).

### miRNA expression analysis by qRT-PCR

For qRT-PCR, susceptible control (3 samples of day-3), susceptible infection (2 samples of day-3), resistant control (1 sample of day-3), and resistant infection (2 samples of day-3) RNA samples were used. cDNA synthesis was performed using miScript II RT Kit (Qiagen) according to the manufacturer’s protocol. Briefly, 1 μg of total RNA was combined with 4 μL of 5 × miScript HiSpec Buffer, 2 μL of 10 × miScript Nucleics Mix, 2 μL of miScript Reverse Transcriptase Mix, and RNase-free water up to 20 μL. The tube was incubated at 37 °C for 60 min and at 95 °C for 5 min to inactivate miScript Reverse Transcriptase Mix and kept on ice. Next, 20 μL of reverse-transcription reaction mixtures were diluted with 130 μL of RNase-free water. The synthesized cDNA was used as a template for qRT-PCR. miScript SYBR Green PCR Kit (Qiagen) was used to determine miRNA expression in LightCycler 96 system (Roche, Indianapolis, IN, USA) according to the manufacturers’ protocol. In brief, for 25 μL of reaction mix, the following components were added: 12.5 μL of 2 × QuantiTect SYBR Green PCR Master Mix, 2.5 μL of 10 μM forward primer, 2.5 μL of 10 × universal primer, 2.5 μL of template cDNA, and RNase-free water up to 25 μL. The cycling conditions were as follows: 95 °C for 15 min to activate as the initial step, followed by 45 cycles of 94 °C for 15 s, 55 °C for 30 s, and 70 °C for 30 s. Gene expression was calculated using the 2^−ΔΔCt^ method after normalization with U1A (5′-CTGCATAATTTGTGGTAGTGG-3′) [35]. All qRT-PCRs were performed in triplicate.

### Statistical analysis

Statistical analysis was performed using SPSS 25.0 software (IBM, Chicago, IL, USA). Data are expressed as mean values ± SEM. Statistical comparisons were performed using Student’s *t*-test for two-group comparisons, and the level of statistically significant difference was set at *p* < 0.05.

## Results

### Exosomal small RNA analysis

**Figure 1:**
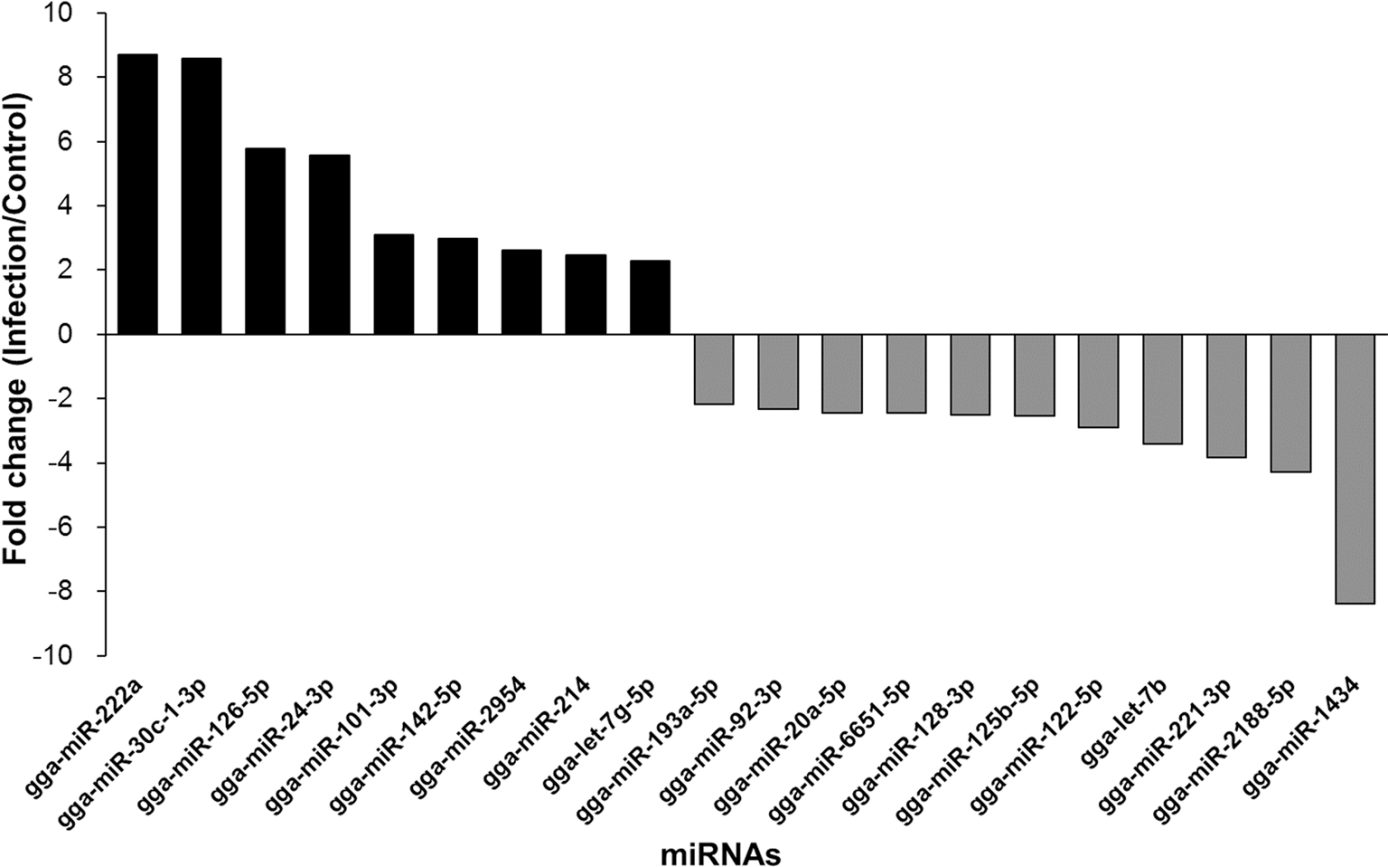
**Differentially expressed miRNA analysis.** Fold-change of 20 miRNAs in the control and avian influenza virus-infected samples. Statistical analysis was performed using fold-change, and significant results were selected on conditions of |FC|≥ 2 and exactTest raw *p*-value < 0.05.

**Figure 2:**
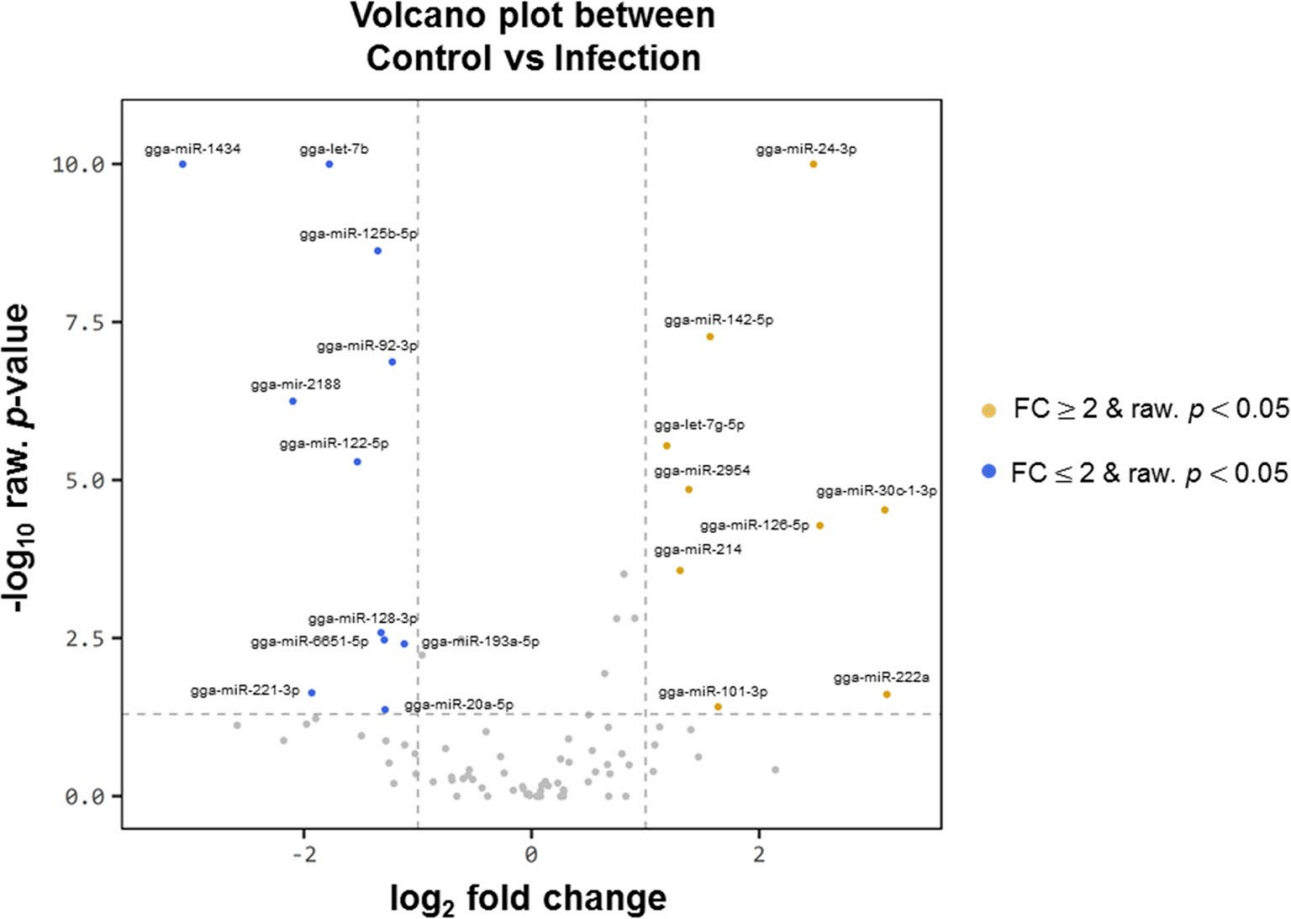
**Volcano plot of expression level of two groups.** X-axis, log_2_ fold-change; Y-axis, −log_10_ *p*-value. Yellow dots indicate FC ≥ 2 and raw *p* < 0.05; blue dot indicate FC ≤  −2 and raw *p* < 0.05.

**Figure 3:**
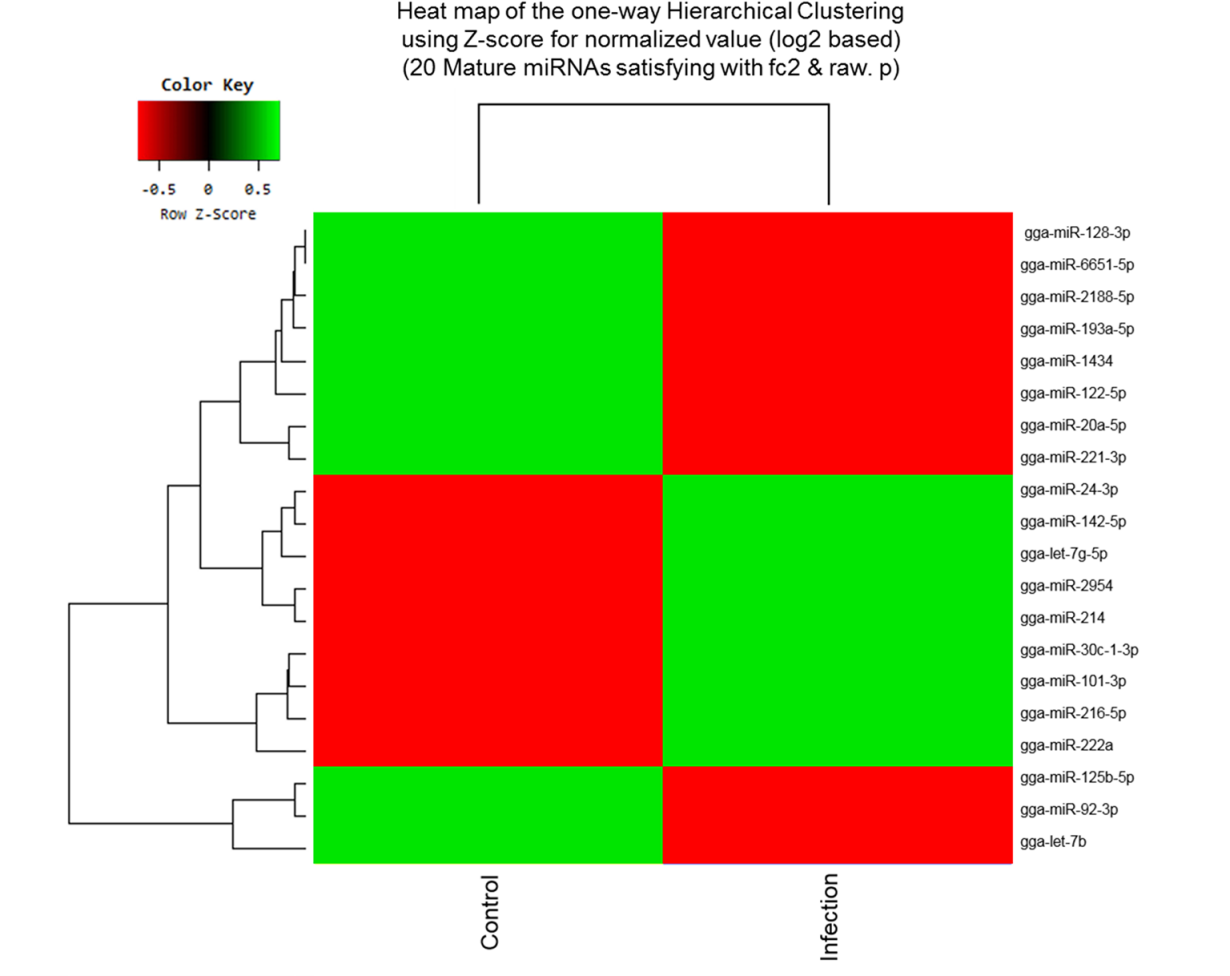
**Hierarchical clustering analysis of 20 miRNAs using R program.** This analysis was conducted using the Euclidean method and complete linkage. The red colour box indicates the control and the light blue colour box indicates infected chickens. Z-score is the estimated coefficient of variation divided by its standard error.

Exosomes were purified from the serum of resistant and susceptible Ri chickens at day 3 post-infection. The size and markers of exosomes were identified (see Additional file 4). Small RNA sequencing was conducted on resistant Ri chickens. In the control sample (Non-infected Ri chickens), 61 116 319 reads were produced, and total read bases were 3.1 Gbp (Table 1). In HPAIV-infected samples, 54 035 519 reads and 2.8 Gbp of read bases were produced. The GC content of control was 38.63% and the ratio of bases with Phred quality score ≥ 30 (Q30) was 90.99%. The GC content of infected chickens was 40.10% and the Q30 was 92.53%. Additional file 5 shows the read length distribution of each sample. In the control, a read length of approximately 17–20 bp was more abundant, whereas the read length was evenly distributed in infected chickens. For control and infected samples, final processed reads were sequentially aligned to the reference genome and the miRBase v21 and ncRNA databases to classify the known miRNAs and other types of RNA, such as tRNA, snoRNA, snRNA, and Piwi-interacting RNA (piRNA) (see Additional file 6). In the control, genome sequences are the largest part as a 92.56% and known miRNA, novel miRNA, snoRNA, snRNA, rRNA, tRNA account for 0.05%, 1.21%, 0.15%, 18.42%, 5.44%, and 0.03%, respectively. Also, genome sequences of the infected samples comprised the largest part (94.1%) and known miRNA, novel miRNA, snoRNA, snRNA, rRNA, and tRNA accounted for 0.03%, 0.32%, 0.04%, 6.46%, 2.25%, and 0.02%, respectively. To predict the known and novel miRNA, unique clustered reads were aligned against the reference genome and precursor miRNAs separately. Novel miRNAs are predicted from mature, star and loop sequence according to the RNAfold algorithm using miRDeep2. To detect known and novel miRNAs, miRDeep2 estimated their abundance (Table 1). In the control, among total 46 317 909 reads, 84.78% (39 269 083 reads) were mapped and 15.22% (7 048 826 reads) were unmapped reads. In infected chickens, among 47 254 454 reads, 90.21% (42 628 445 reads) were mapped and 9.79% (4 626 009 reads) were unmapped reads. We also investigated differentially expressed miRNA analysis by the read count value of mature miRNAs (see Additional file 7). A total of 20 miRNAs showed significantly different fold-change (Figure 1). Among 20 miRNAs, nine miRNAs were up-regulated and 11 miRNAs were down-regulated in infected chickens compared to those in the control. In particular, gga-miR-222a was the highest, with a fold-change of 8.69, and gga-miR-1434 was the lowest, with fold-change of −8.36. gga-miR-222a, gga-miR-30c-1-3p, gga-miR-126-5p, gga-miR-24-3p, gga-miR-101-3p, gga-miR-142-5p, gga-miR-2954, gga-miR-214, and gga-let-7g-5p were up-regulated in infected samples compared with that in the control, whereas gga-miR-193a-5p, gga-miR-92-3p, gga-miR-20a-5p, gga-miR-6651-5p, gga-miR-128-3p, gga-miR-125-5p, gga-miR-122-5p, gga-let-7b, gga-miR-221-3p, gga-miR-2188-5p, and gga-miR-1434 were down-regulated in infected samples compared with those in the control. We also compared the expression levels of miRNAs between control and infected samples using a volcano plot (Figure 2). Log_2_ fold-change and *p*-values obtained from the comparison between the two groups were plotted as a volcano plot. gga-miR-1434 displayed both high fold-change (x-axis) and statistical significance (y-axis). We also conducted hierarchical clustering analysis by the Euclidean method and complete linkage, which clusters similar mature miRNAs and samples by expression level (normalized value) from the differentially expressed miRNA list (Figure 3). Nine up-regulated exosomal miRNAs in the infected chickens were grouped in the generated dendrogram.

**Table 1:** **Raw data statistics**

Sample	Total reads bases	Total reads	Processed reads	Mapped reads	GC (%)	Q20 (%)	Q30 (%)	Known miRNA in sample	Known miRNA in chicken (miRBase v21)
Control	3 116 932 269	61 116 319	46 317 909	26 209 (0.06%)	39	95	91	152	994
Infection	2 755 811 469	54 035 519	47 254 454	16 389 (0.03%)	40	96	93	136	994

Total reads bases = total read × read length. Total read bases indicate the total number of bases sequenced and total reads indicate the total number of reads. Processed reads indicate reads that were trimmed and unwanted sources were deleted. Q20 (%) is the ratio of bases with Phred quality score of ≥ 20. Q30 (%) is the ratio of bases with Phred quality score of ≥ 30.

### Target gene prediction, GO functional enrichment, and Kyoto Encyclopedia of Genes and Genomes (KEGG) pathway analysis

**Figure 4:**
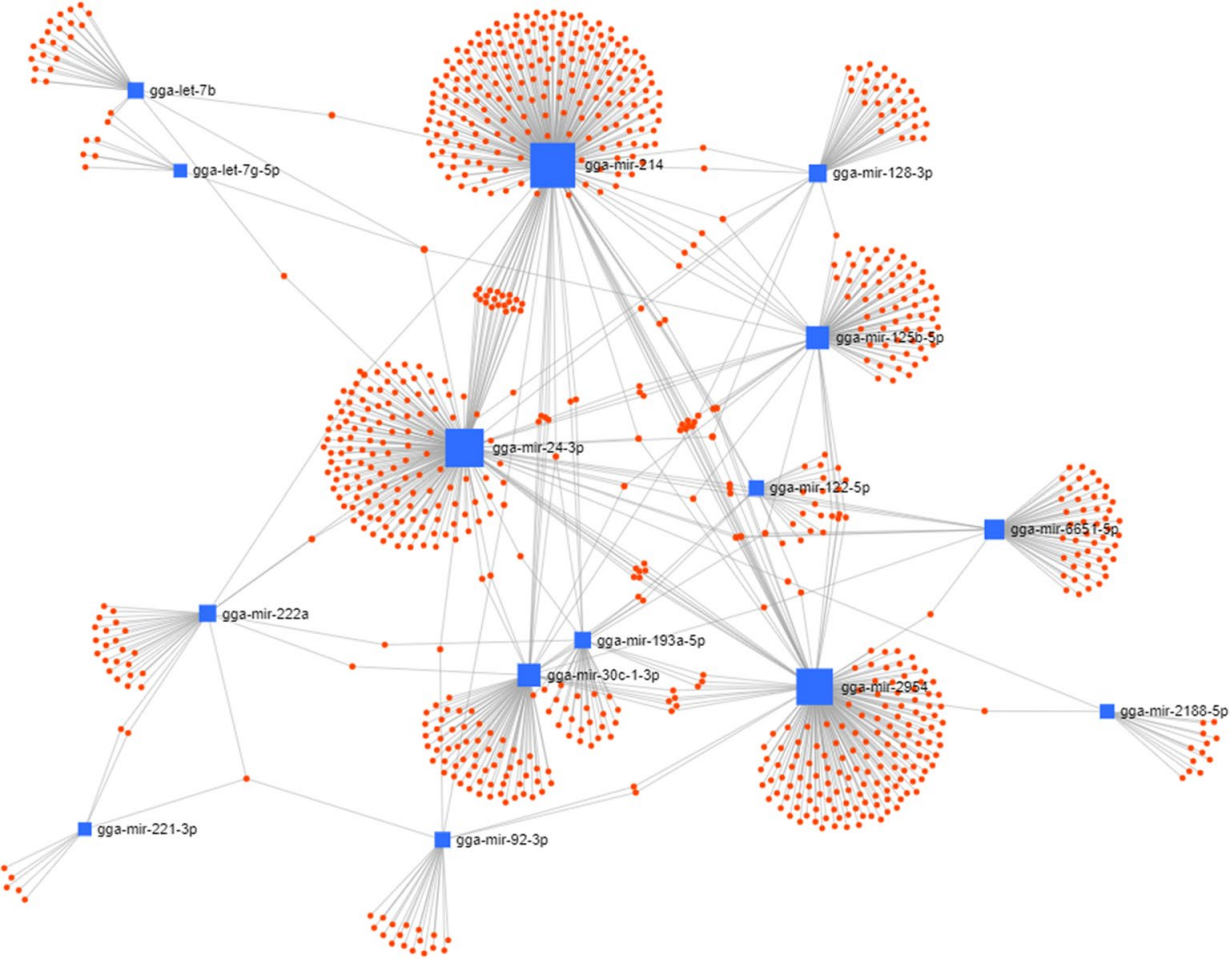
**miRNA/mRNA network analysis.** The interaction of 20 differentially expressed miRNAs and mRNAs target genes was analyzed using miRNet [33] based on miRNA target prediction results by miRanda. The blue squares represent the miRNA and red nodes represent its target genes.

The target genes of 20 miRNAs were predicted using miRDB. Immune-related target genes with score of over 80 were selected (Table 2). In particular, 32 immune-related genes were related to gga-miR-20a-5p, and no immune-related target genes were related to gga-miR-1434. We also conducted GO analysis using gProfiler (see Additional file 8). In KEGG pathway analysis, eight pathways, including erythroblastic leukemia viral oncogene homolog (ErbB) signalling pathway, advanced glycation end-products (AGE)–receptor of AGE (RAGE) signalling pathway in diabetic complications, mitogen-activated protein kinase (MAPK) signalling pathway, focal adhesion, regulation of actin cytoskeleton, insulin signalling pathway, phosphatidylinositol signalling system, and endocytosis, were related to 20 miRNAs (Table 3). In particular, 69 target genes of miRNAs were related with the MAPK signalling pathway, followed by endocytosis (59 genes), focal adhesion (52 genes), and regulation of actin cytoskeleton (52 genes). Furthermore, the miRNA-mRNA target gene interaction of 20 differentially expressed miRNAs was analysed. As shown in Figure 4, several targets genes were shared with more than two miRNAs.

**Table 2:** **Immune-related target genes for miRNAs**

miRNAs	Fold change (infection/control)	Immnue related target genes	Gene symbol	Control read count	Infection read count
gga-let-7b	−3.425194	24	IGDCC3, COL4A1, COL4A2, AGO4, LRIG3, COL1A2, IL13RA1, LRIG1, MAPK6, TGFBR1, IGDCC4, COL3A1, TNFAIP2, WNT9B, SEC14L1, DCLRE1C, ERCC6, CCR6, MAP4K3, EDN1, TBKBP1, MAP3K1, MAP4K4, FGF6	8110	1943
gga-let-7 g-5p	2.2773292	14	IL13RA1, MAPK6, TGFBR1, WNT9B, SEC14L1, DCLRE1C, CCR6, ERCC6, MAP4K3, MAP3K1, EDN1, TBKBP1, MAP4K4, FGF6	146	273
gga-miR-101-3p	3.110288	21	MAP3K13, MAPK6, CD274, MAP3K9, CREB1, MAP3K4, BACH2, MAPK1, NLK, CCR2, PIK3CG, SYNCRIP, SH3GL3, MEF2A, MAPK8, HACD3, MAPKAPK5, NF1, STAT3, CAMK2D, TRAF3	5	13
gga-miR-122-5p	−2.889137	1	GREM2	127	36
gga-miR-125b-5p	−2.550591	8	IRF4, PTPN1, SMURF1, PRDM1, TSTA3, IL17RA, TRAF3IP2, IL13RA1	1001	322
gga-miR-126-5p	5.7802815	17	LIF, TRAF3, FGFRL1, FGF12, C6, C1S, FGF7, IL17A, DENND1B, FGF14, CREB1, MAPK10, GATA3, SARM1, ERAP1, GDF9, JAG1	6	29
gga-miR-128-3p	−2.500261	26	JAG1, GAB1, MAPK8IP3, NF1, ZFP36L1, DAB2IP, SH3BGRL2, NCF1, MAPK14, MAPKBP1, C1S, IL9R, WNT2, MAP2K3, ITCH, DENND1B, TSC1, SH3RF1, SETX, FGF14, TRIL, IL17RA, PTPRC, RAB20, JAK2, PRDM11	52	17
gga-miR-142-5p	2.9630095	7	IL8L1, ITCH, TAB3, SFPQ, MAP4K4, FGF10, BMP15	69	168
gga-miR-1434	−8.369591	0		103	10
gga-miR-193a-5p	−2.172781	1	PDCD1LG2	69	26
gga-miR-20a-5p	−2.441626	32	MAP3K2, IL1RAPL1, CD274, PDCD1LG2, TGFBR2, BPIFB3, SOS1, ITCH, GAB1, MAP3K14, IPO7, MAPK1, MAP3K9, PRDM11, TNFRSF21, FAM3C, CREB5, GPI, TGFBRAP1, SMAD6, AKT3, TOLLIP, SH3PXD2A, SH3GLB2, MAP3K5, PKD1, CAMK2D, MAP1LC3A, TNFSF11, CREB1, SAMHD1, IL17RD	24	8
gga-miR-214	2.464608	16	SOS2, IRF8, MAPK1, EPHB6, TAB3, FGF14, LIF, DMAP1, DNAJA3, CD8A, NF1, PIK3CG, SH3PXD2A, TRAF3, LACC1, CCR2	37	75
gga-miR-2188-5p	−4.285245	4	FOSB, FGF20, PAK3, JUN	84	16
gga-miR-221-3p	−3.818655	8	PRDM1, ANKHD1, FOS, TAB2, CSF1R, ERBB4, IPO7, SMURF2	19	4
gga-miR-222a	8.6921924	8	PRDM1, ANKHD1, FOS, CSF1R, TAB2, ERBB4, IPO7, SMURF2	1	8
gga-miR-24-3p	5.5548592	2	MAPKAP1, OTUD7B	51	233
gga-miR-2954	2.6033041	4	TJP2, SYK, NFIL3, CD274	50	107
gga-miR-30c-1-3p	8.5887224	7	SCG2, LIFR, TAB3, TRAF2, MAP3K3, TOLLIP, SEC14L1	3	22
gga-miR-6651-5p	−2.452306	4	HIF1A, ELMOD2, LTA4H, AvBD8	51	17
gga-miR-92-3p	−2.335071	15	SMURF1, MAP2K4, DENND1B, RORA, MAP3K20, G3BP2, SH3PXD2A, TSC1, PIK3CD, GREM2, GSN, LRRK2, DNAJB9, TRAF3, UBASH3B	703	247

**Table 3:** **KEGG pathway analysis**

KEGG	Pathway	Gene Count	*p*-value
KEGG:04012	ErbB signalling pathway	29	5.87E−04
KEGG:04933	AGE–RAGE signalling pathway in diabetic complications	31	8.56E−04
KEGG:04010	MAPK signalling pathway	69	1.05E−03
KEGG:04510	Focal adhesion	52	1.15E−03
KEGG:04810	Regulation of actin cytoskeleton	52	6.02E−03
KEGG:04910	Insulin signalling pathway	36	7.88E−03
KEGG:04070	Phosphatidylinositol signalling system	30	1.02E−02
KEGG:04144	Endocytosis	59	1.36E−02

### Validation of miRNA expression by qRT-PCR

**Figure 5:**
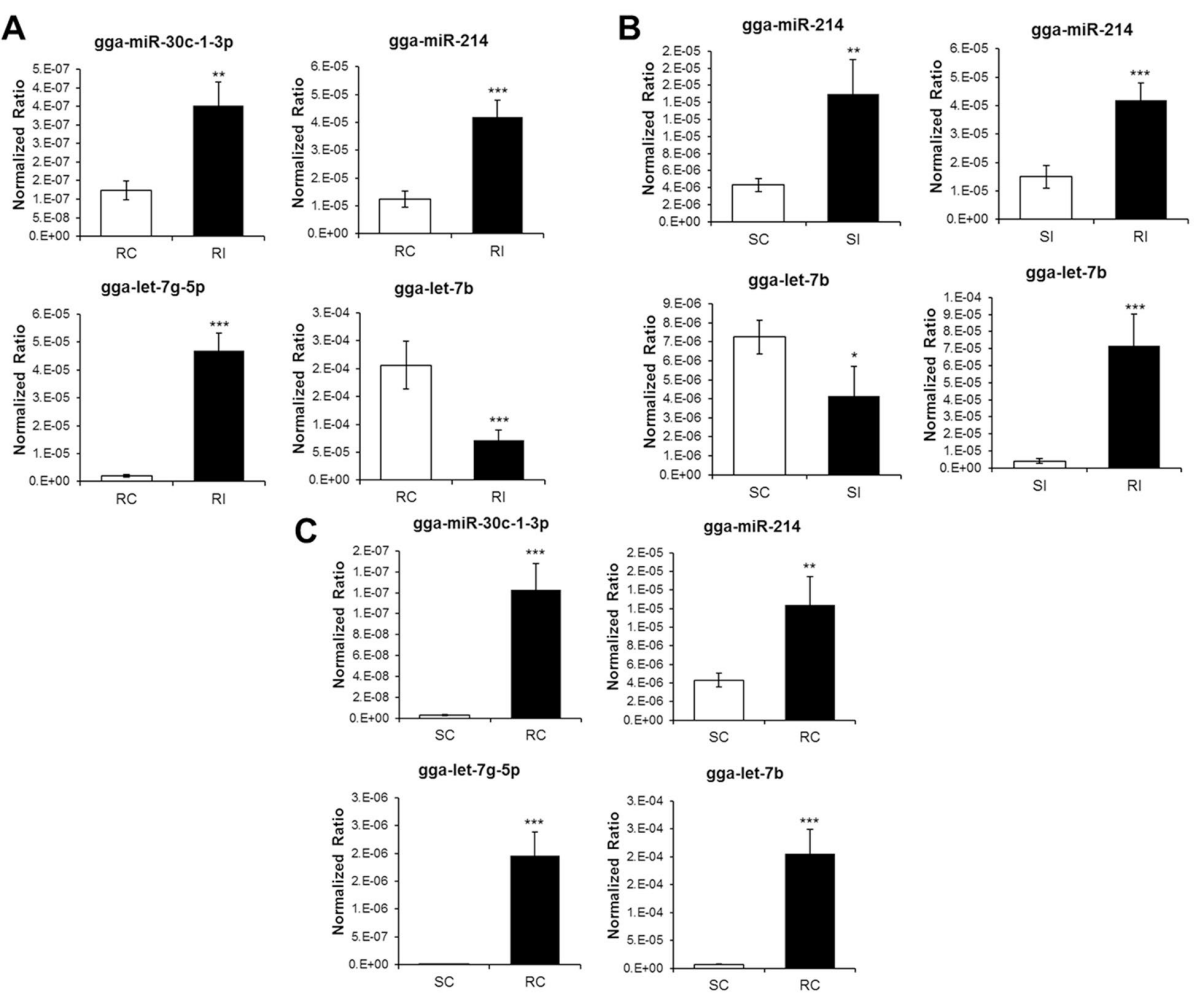
**qRT-PCR of exosomal miRNA.** **A** Validation of exosomal miRNAs expression in resistant Ri chickens by qRT-PCR. **B** Expression of exosomal miRNAs between resistant and susceptible Ri chickens. RC, resistant control; RI, resistant infection; SC, susceptible control; SI, susceptible infection. Relative quantitation data are represented as mean ± SEM normalized to U1A using the 2^−ΔΔCt^ method. Data are expressed as mean ± SEM of three independent experiments: *p < 0.05, **p < 0.01, and ***p < 0.001.

qRT-PCR was conducted using four miRNAs, selected on the basis of significant difference of > 2.0 or < 2.0 fold-change between control and infected chickens, to validate sequencing results (Figure 5A). The four miRNAs were selected based on read count, the number of immune-related genes, and functions in immune system. The expression levels of gga-miR-30c-1-3p, gga-miR-214, and gga-let-7g-5p were up-regulated in infected resistant chickens compared with those in the control by 2.85, 3.37, and 23.91 fold-change, respectively. However, the expression of gga-let-7b was down-regulated in infected chickens (0.34 fold-change). qRT-PCR results of the four miRNAs were positively correlated with sequencing results. The expression levels of gga-miR-214 and gga-let-7b were also evaluated in resistant and susceptible Ri chickens (Figure 5B). The expression level of gga-miR-214 was up-regulated in susceptible infected chickens compared with that in the control (3.48 fold-change). However, the expression level of gga-let-7b was down-regulated in susceptible infected chickens compared with that in the control (0.57 fold-change). The expression patterns of gga-miR-214 and gga-let-7b were similar in susceptible and resistant chickens. Moreover, the expression levels of gga-miR-214 and gga-let-7b were higher (2.79 and 17.18 fold-change, respectively) in resistant chickens than in susceptible chickens. Furthermore, the expression of four miRNAs was compared between susceptible and resistant mock control (Figure 5C). The expression levels of gga-miR-214, gga-miR-30c-1-3p, gga-let-7g-5p, and gga-let-7b were higher in resistant than susceptible control chicken (2.88, 45.67, 180.39, and 28.34 fold-change, respectively).

## Discussion

In this study, we report exosomal miRNA analysis of avian influenza virus infected chickens. The Vietnamese AIV-resistant Ri chickens were selected by *Mx* and *BF2* genotyping and infected with H5N1 HPAIV. Then, exosomes were isolated from the serum, and miRNA was analysed by small RNA sequencing and qRT-PCR.

Exosomes are formed by various molecules, such as proteins, lipids, DNA, mRNA, and miRNA, that are contained in endosomes through endocytosis. Then, endosomes called MVBs fuse with the cell membrane and are released into the extracellular space [36]. Released exosomes containing miRNA are delivered to other cells through biological fluids and regulate the gene expression of recipient cells; however, they are not randomly packaged in exosomes [37–40].

In this study, 20 exosomal miRNAs exhibited significantly different expression between control and infected resistant Ri chickens (Figure 1). Previous studies have reported differentially expressed miRNA in chickens and humans. The expression of gga-miR-6651-5p increases in Marek’s disease virus infected-chickens [41]; gga-miR-92-3p and gga-miR-214 are abundant in AIV H9N2-infected chicken embryo fibroblasts [42]; gga-miR-2954 is up-regulated in reticuloendotheliosis virus-infected chickens [43]; gga-miR-222a, gga-miR-125b-5p, and gga-miR-128-3p are down-regulated in H9N2-infected chicken embryo fibroblasts [42]. Likewise, in our study, gga-miR-125b and gga-miR-128-3p were down-regulated in H5N1-infected chickens. In another study, miR-126-5p inhibited human cervical cancer progression, thus, regulating the apoptosis of cancer cells [44]. gga-miR-101-3p is up-regulated in *Mycoplasma gallisepticm*-infected chickens [45]. gga-let-7b and miR-128 reduce cell growth and division during skeletal muscle development in sex-linked dwarf chickens [46]. gga-let-7b is involved in signalling pathways, such as MAPK, TGF-β, Notch, Wnt, mTOR, cell cycle, p53, and Janus-activated kinase (JAK)–signal transducers and activators of transcription (STAT) pathways [47]. Human miR-20a-5p inhibits cell proliferation and induces apoptosis in SH-SY5Y cells [48], and its down-regulation induces cell apoptosis to remove mycobacterial cells through targeting JNK2 in human macrophages [49]. Therefore, we suggest that 19 differentially expressed exosomal miRNAs from AI-infected chickens regulate immune response.

We analysed immune-related target genes (Table 2). Various immune-related genes, such as genes encoding signalling pathway molecules, cytokines, and chemokines, were found to be miRNA target genes. In particular, in KEGG pathway analysis, the highest number of target genes (69 gene count) were related with the MAPK signalling pathway (Table 3). The MAPK signalling pathway plays important roles in the immune system and also in cell proliferation, differentiation, migration, and apoptosis [50]. MAPK signalling pathway was activated by H5N1 AIV [51–53]. Several molecules of the MAPK pathway are differently regulated in two inbred necrotic enteritis-afflicted chicken lines, 6.3 and 7.2 [54]. Furthermore, the MAPK pathway plays an important role in virus replication in chicken macrophages infected with H9N2 AIV [55]. MAPK signalling pathway was followed by Endocytosis (59 gene count). As a first step of AIV infection, AIV enters the cells by endocytosis [56]. The next KEGG pathway was Focal adhesion and regulation of actin cytoskeleton (52 gene count). Focal adhesion kinase was activated during AIV infection by inducing actin rearrangement [57]. Therefore, we suggest that the target genes of 20 exosomal miRNAs regulate pro-inflammatory signalling pathway against AIV infection and life cycle of AIV.

We also compared the expression of gga-miR-214 and gga-let-7b between resistant and susceptible Ri chickens (Figure 4B). The expression patterns between control and infected chickens were the same, but the expression levels were higher in resistant than in susceptible chickens. Therefore, we suggest that the copy number of miRNAs is higher in the exosomes of resistant Ri chickens than in the exosomes of susceptible Ri chickens. Accordingly, regulation of gene expression by exosomal miRNAs may be higher in resistant Ri chickens. Hence, resistant Ri chickens could respond more actively than susceptible Ri chickens against AIV infection.

So far, several studies have analysed miRNA expression patterns on AIV infection [45, 58–61]. However, there have limited studies on the exosomes in chickens until now.

Taken together, this study provides an insight into the exosomal miRNA expression pattern in AIV-resistant and -susceptible chickens and the mechanism by which they regulate target genes toward HPAIV infection.

In summary, we have, for the first time, analysed the exosomal miRNA expression in H5N1 HPAIV-infected chickens by small RNA sequencing and qRT-PCR. A total of 20 miRNAs were differentially expressed in exosomes of control and infected resistant Ri chickens. Interestingly, most of the target genes were related with the MAPK signalling pathway. This study improves our understanding of the host immune response, particularly with respect to exosomal miRNA expression, against H5N1 HPAIV infection.

## Supplementary Information

**Additional file 1. Number of Ri chicken samples in each group**

**Additional file 2. Sequencing analysis of Mx in avian influenza virus-resistant and -susceptible Ri chickens**

**Additional file 3. Sequences of primers for qRT-PCR analysis**

**Additional file 4. Characterization of purified exosomes**. (A) Particle size distribution measured by Nanoparticle Analyzer. (B) Western blotting of exosomes with exosomal marker CD81.

**Additional file 5. Read length distribution of control and infection samples. Generally mature miRNAs are 20 ~ 25 nt in length**.

**Additional file 6. Bar plot of RNA composition in the control and avian influenza virus-infected samples.** Final processed reads were aligned to small RNAs (≤ 50; piRNA) of the database using bowtie [62] and other small RNAs (≥ 50 nt; tRNA, snoRNA, etc.) of the database using bowtie2, which assigned a result of ≥ 90% coverage to the corresponding RNA.

**Additional file 7. Raw small RNA sequencing data**.

**Additional file 8. Gene ontology analysis.** (A) Biological process (B) Cellular component (C) Molecular function. Target categorized in specific functional groups according to gene ontology using Fisher’s exact test (*p* < 0.01).

## Data Availability

All data generated or analyzed during this study are included in this published article and its additional information files.

## Abbreviations

miRNA: Micro RNA
AIV: Avian influenza viruses
HPAIV: Highly pathogenic avian influenza virus
MAPK: Mitogen-activated protein kinase
MVBs: Multivesicular bodies
ILVs: Intraluminal vesicles
ncRNAs: Non-coding RNAs
MHC: Major histocompatibility complex
SPF: Specific-pathogen-free
snRNA: Small nuclear RNA
snoRNA: Small nucleolar RNA
qRT-PCR: Quantitative real-time PCR
KEGG: Kyoto Encyclopedia of Genes and Genomes
ErbB: Erythroblastic leukemia viral oncogene homolog
AGE–RAGE signalling pathway: Advanced glycation end-products–receptor of AGE signalling pathway
JAK–STAT signalling pathways: Janus-activated kinase–signal transducers and activators of transcription pathways
