# Supplementary material for: Exosomal miRNA profiling from H5N1 avian influenza virus-infected chickens
Source: Vet Res. 2021 Mar 3;52:36. doi: 10.1186/s13567-021-00892-3 (PMC7931527; doi:10.1186/s13567-021-00892-3)
Supplement: Supplementary file 1 — Additional file 1. Number of Ri chicken samples in each group [file 13567_2021_892_MOESM1_ESM.docx]

**Table S1.** Number of Ri chicken samples in each group

| Sample | Genotype | | | | | | | |
| --- | --- | --- | --- | --- | --- | --- | --- | --- |
| Ri  chickens (40) | Resistant AA/Mx^++^ | | | | Susceptible GG/Mx^−−^ | | | |
|  | Control | | HPAIV infection | | Control | | HPAIV infection | |
|  | Day 1 | Day 3 | Day 1 | Day 3 | Day 1 | Day 3 | Day 1 | Day 3 |
|  | 5 | 5 | 5 | 5 | 5 | 5 | 5 | 5 |
