# Supplementary material for: Exosomal miRNA profiling from H5N1 avian influenza virus-infected chickens
Source: Vet Res. 2021 Mar 3;52:36. doi: 10.1186/s13567-021-00892-3 (PMC7931527; doi:10.1186/s13567-021-00892-3)
Supplement: Supplementary file 2 — Additional file 2. Sequencing analysis of Mx in avian influenza virus-resistant and -susceptible Ri chickens [file 13567_2021_892_MOESM2_ESM.docx]

**
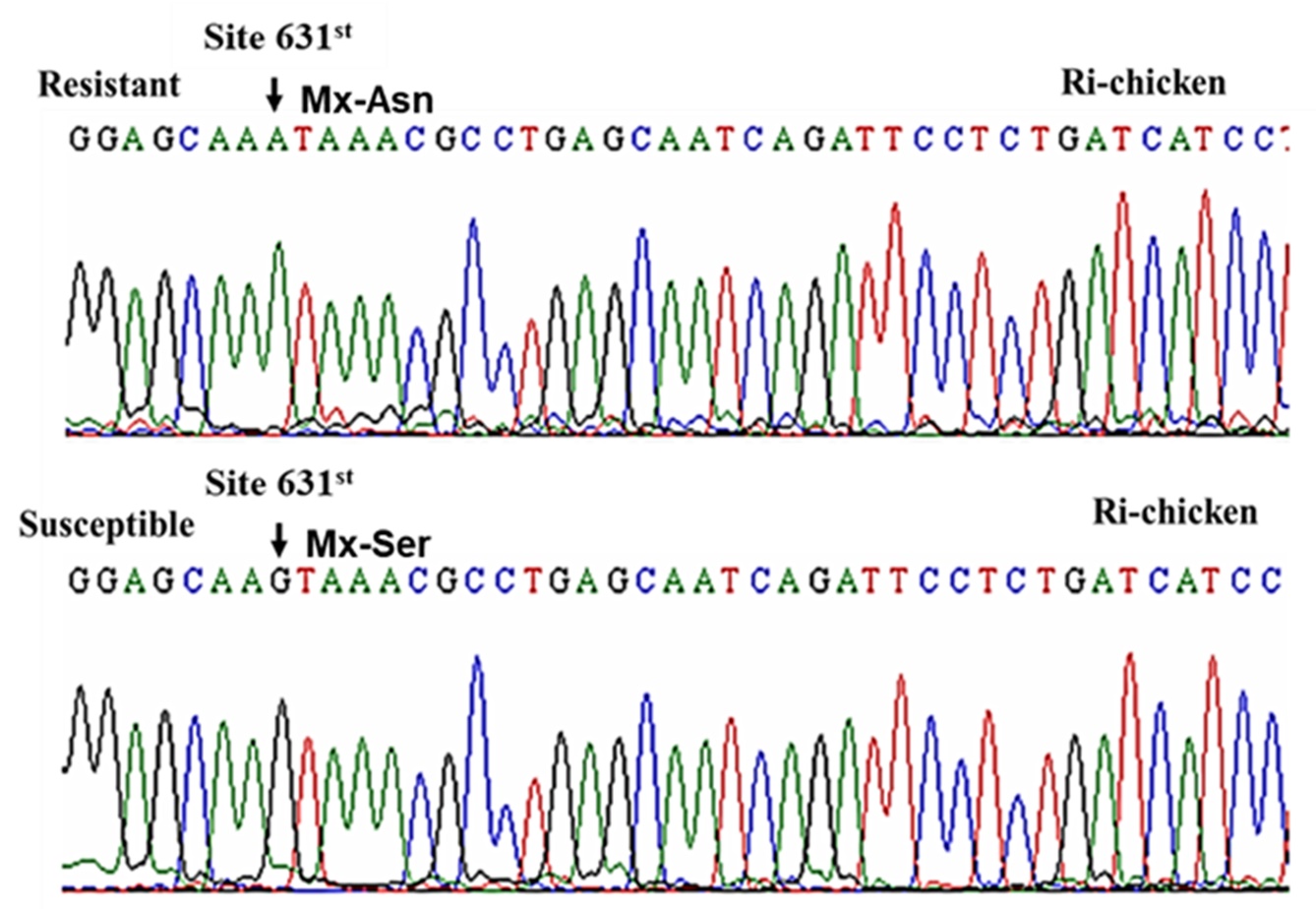
**

**Figure S1.** Sequencing analysis of *Mx* in avian influenza virus-resistant and -susceptible Ri chickens.
