# Supplementary material for: Exosomal miRNA profiling from H5N1 avian influenza virus-infected chickens
Source: Vet Res. 2021 Mar 3;52:36. doi: 10.1186/s13567-021-00892-3 (PMC7931527; doi:10.1186/s13567-021-00892-3)
Supplement: Supplementary file 3 — Additional file 3. Sequences of primers for qRT-PCR analysis [file 13567_2021_892_MOESM3_ESM.docx]

**Table S2.** Sequences of primers for qRT-PCR analysis.

| **miRNAs** | **Sequences (5′- 3′)** |
| --- | --- |
| **gga-miR-30c-1-3p** | TGGGAGAGGATTGTTTACGCC |
| **gga-miR-214** | ACAGCAGGCACAGACAGGCAG |
| **gga-let-7g-5p** | TGAGGTAGTAGTTTGTACAGT |
| **gga-let-7b** | TGAGGTAGTAGGTTGTGTGGTT |
| **U1A** | CTGCATAATTTGTGGTAGTGG |
