# Supplementary material for: Exosomal miRNA profiling from H5N1 avian influenza virus-infected chickens
Source: Vet Res. 2021 Mar 3;52:36. doi: 10.1186/s13567-021-00892-3 (PMC7931527; doi:10.1186/s13567-021-00892-3)
Supplement: Supplementary file 4 — Additional file 4. Characterization of purified exosomes. (A) Particle size distribution measured by Nanoparticle Analyzer. (B) Western blotting of exosomes with exosomal marker CD81. [file 13567_2021_892_MOESM4_ESM.docx]

**
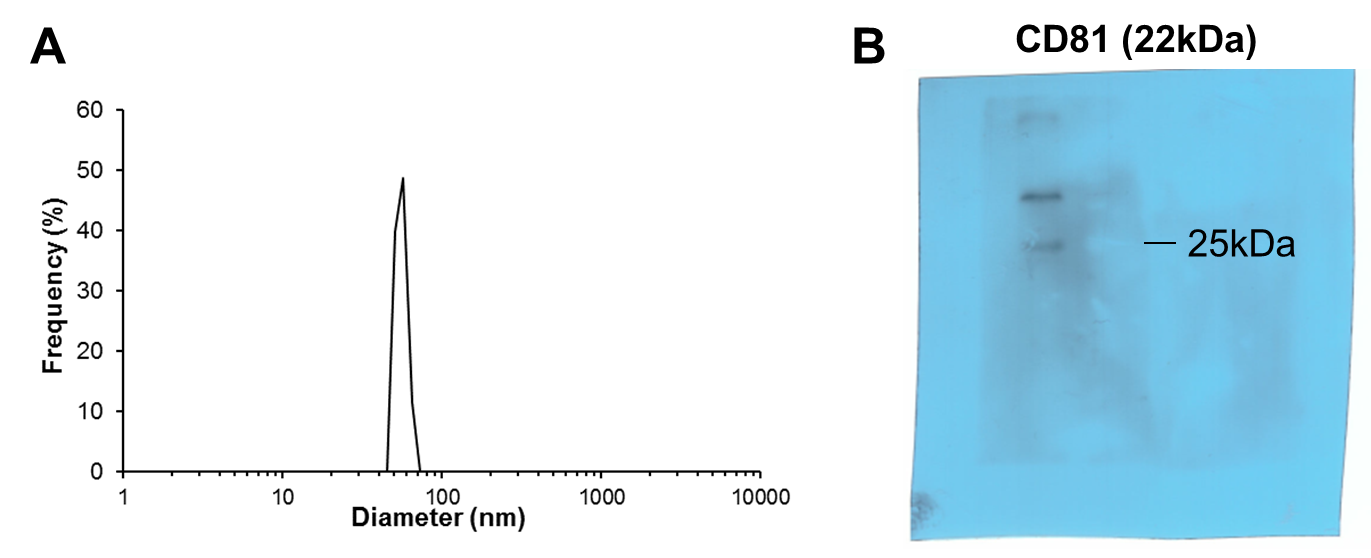
**

**Figure S2.** Characterization of purified exosomes. (A) Particle size distribution measured by Nanoparticle Analyzer. (B) Western blotting of exosomes with exosomal marker CD81.
