# Supplementary material for: Exosomal miRNA profiling from H5N1 avian influenza virus-infected chickens
Source: Vet Res. 2021 Mar 3;52:36. doi: 10.1186/s13567-021-00892-3 (PMC7931527; doi:10.1186/s13567-021-00892-3)
Supplement: Supplementary file 5 — Additional file 5. Read length distribution of control and infection samples. Generally mature miRNAs are 20 ~ 25 nt in length. [file 13567_2021_892_MOESM5_ESM.docx]

**
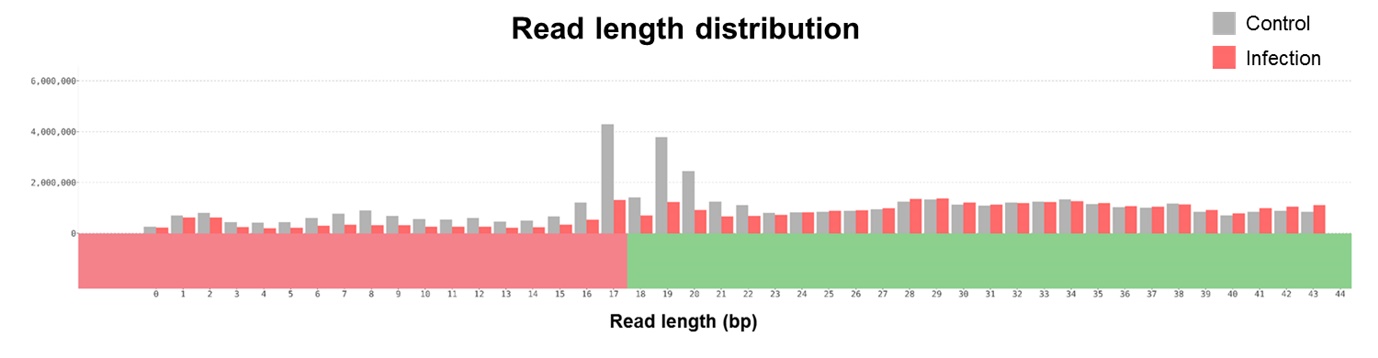
**

**Figure S3.** Read length distribution of control and infection samples. Generally mature miRNAs are 20~25 nt in length.
