# Supplementary material for: Exosomal miRNA profiling from H5N1 avian influenza virus-infected chickens
Source: Vet Res. 2021 Mar 3;52:36. doi: 10.1186/s13567-021-00892-3 (PMC7931527; doi:10.1186/s13567-021-00892-3)
Supplement: Supplementary file 6 — Additional file 6. Bar plot of RNA composition in the control and avian influenza virus-infected samples. Final processed reads were aligned to small RNAs (≤ 50; piRNA) of the database using bowtie [62] and other small RNAs (≥ 50 nt; tRNA, snoRNA, etc.) of the database using bowtie2, which assigned a result of ≥ 90% coverage to the corresponding RNA. [file 13567_2021_892_MOESM6_ESM.docx]

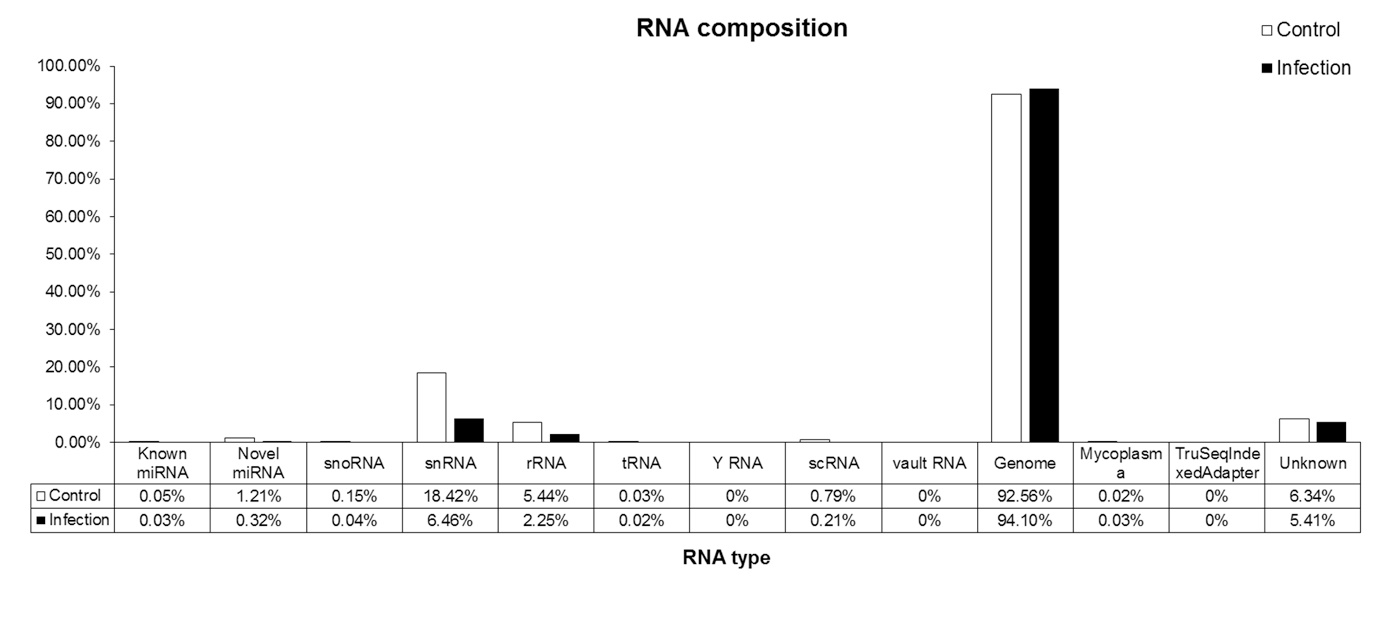


**Figure S4.** Bar plot of RNA composition in the control and avian influenza virus-infected samples. Final processed reads were aligned to small RNAs (≤50; piRNA) of the database using bowtie (<http://bowtie-bio.sourceforge.net/index.shtml>) and other small RNAs (≥50 nt; tRNA, snoRNA, etc.) of the database using bowtie2, which assigned a result of ≥90% coverage to the corresponding RNA.
