# Supplementary material for: Exosomal miRNA profiling from H5N1 avian influenza virus-infected chickens
Source: Vet Res. 2021 Mar 3;52:36. doi: 10.1186/s13567-021-00892-3 (PMC7931527; doi:10.1186/s13567-021-00892-3)
Supplement: Supplementary file 7 — Additional file 7. Raw small RNA sequencing data. [file 13567_2021_892_MOESM7_ESM.docx]

**Table S3.** Raw small RNA sequencing data

| **Mature ID** | **Mature Accession** | **RC**  **RPM** | **RI**  **RPM** | **RI/RC.fc** | **RI/RC.**  **logCPM** | **RI/RC.**  **raw.pval** | **RI/RC.**  **bh.pval** | **N RC** | **N RI** | **RC Read Count** | **RI Read Count** |
| --- | --- | --- | --- | --- | --- | --- | --- | --- | --- | --- | --- |
| [gga-miR-1434](http://www.mirbase.org/cgi-bin/query.pl?terms=gga-miR-1434) | MIMAT0007295 | 3748.58973 | 565.706851 | -8.369591001 | 11.29592037 | 2.801E-12 | 9.89686E-11 | 12.0526859 | 8.97581321 | 103 | 10 |
| [gga-miR-17-3p](http://www.mirbase.org/cgi-bin/query.pl?terms=gga-miR-17-3p) | MIMAT0001115 | 291.1526 | 56.570685 | -6.001549659 | 8.185190511 | 0.075793071 | 0.259163403 | 8.37021238 | 5.67944595 | 8 | 1 |
| [gga-miR-451](http://www.mirbase.org/cgi-bin/query.pl?terms=gga-miR-451) | MIMAT0003775 | 218.36445 | 56.570685 | -4.526480702 | 7.948558489 | 0.131529066 | 0.35293643 | 7.95662749 | 5.67944595 | 6 | 1 |
| [gga-miR-2188-5p](http://www.mirbase.org/cgi-bin/query.pl?terms=gga-miR-2188-5p) | MIMAT0016372 | 3057.102304 | 905.130961 | -4.28524484 | 11.14327595 | 5.61516E-07 | 8.50296E-06 | 11.75857961 | 9.65281019 | 84 | 16 |
| [gga-miR-29a-3p](http://www.mirbase.org/cgi-bin/query.pl?terms=gga-miR-29a-3p) | MIMAT0001096 | 363.94075 | 113.14137 | -3.937702412 | 8.483829381 | 0.072711657 | 0.256914522 | 8.6912682 | 6.66530143 | 10 | 2 |
| [gga-miR-29c-3p](http://www.mirbase.org/cgi-bin/query.pl?terms=gga-miR-29c-3p) | MIMAT0001183 | 363.94075 | 113.14137 | -3.937702412 | 8.483829381 | 0.072711657 | 0.256914522 | 8.6912682 | 6.66530143 | 10 | 2 |
| [gga-miR-221-3p](http://www.mirbase.org/cgi-bin/query.pl?terms=gga-miR-221-3p) | MIMAT0001108 | 691.487426 | 226.28274 | -3.818654563 | 9.228956445 | 0.023156585 | 0.106721652 | 9.61561345 | 7.65817683 | 19 | 4 |
| [gga-miR-193b-5p](http://www.mirbase.org/cgi-bin/query.pl?terms=gga-miR-193b-5p) | MIMAT0026629 | 509.517051 | 169.712055 | -3.72723295 | 8.871705608 | 0.058628571 | 0.221951018 | 9.1756975 | 7.24551811 | 14 | 3 |
| [gga-let-7b](http://www.mirbase.org/cgi-bin/query.pl?terms=gga-let-7b) | MIMAT0001102 | 295155.9486 | 109916.8411 | -3.425193887 | 17.72154516 | 6.9788E-17 | 3.69876E-15 | 18.35133626 | 16.57510182 | 8110 | 1943 |
| [gga-miR-122-5p](http://www.mirbase.org/cgi-bin/query.pl?terms=gga-miR-122-5p) | MIMAT0001190 | 4622.047531 | 2036.544663 | -2.889137296 | 11.83240901 | 5.13795E-06 | 6.05137E-05 | 12.35480589 | 10.82173917 | 127 | 36 |
| [gga-miR-148a-3p](http://www.mirbase.org/cgi-bin/query.pl?terms=gga-miR-148a-3p) | MIMAT0001120 | 509.517051 | 226.28274 | -2.82095589 | 8.941680416 | 0.111029597 | 0.318084792 | 9.1756975 | 7.65817683 | 14 | 4 |
| [gga-miR-125b-5p](http://www.mirbase.org/cgi-bin/query.pl?terms=gga-miR-125b-5p) | MIMAT0001105 | 36430.46912 | 18215.76059 | -2.550590626 | 14.81639462 | 2.34429E-09 | 6.21236E-08 | 15.33310695 | 13.9820229 | 1001 | 322 |
| [gga-miR-128-3p](http://www.mirbase.org/cgi-bin/query.pl?terms=gga-miR-128-3p) | MIMAT0001123 | 1892.491902 | 961.701646 | -2.500261382 | 10.65514064 | 0.002576311 | 0.016064054 | 11.06695811 | 9.7401676 | 52 | 17 |
| [gga-miR-6651-5p](http://www.mirbase.org/cgi-bin/query.pl?terms=gga-miR-6651-5p) | MIMAT0025751 | 1856.097827 | 961.701646 | -2.452306065 | 10.63608299 | 0.003376869 | 0.018839372 | 11.03895692 | 9.7401676 | 51 | 17 |
| [gga-miR-20a-5p](http://www.mirbase.org/cgi-bin/query.pl?terms=gga-miR-20a-5p) | MIMAT0001111 | 873.457801 | 452.565481 | -2.441626418 | 9.646936509 | 0.042538843 | 0.173427592 | 9.95226526 | 8.65460128 | 24 | 8 |
| [gga-miR-29b-1-5p](http://www.mirbase.org/cgi-bin/query.pl?terms=gga-miR-29b-1-5p) | MIMAT0026488 | 545.911126 | 282.853425 | -2.429711124 | 9.068229825 | 0.133183558 | 0.35293643 | 9.27506686 | 7.97867577 | 15 | 5 |
| [gga-miR-6594-5p](http://www.mirbase.org/cgi-bin/query.pl?terms=gga-miR-6594-5p) | MIMAT0025688 | 218.36445 | 113.14137 | -2.383956558 | 8.077314069 | 0.298737053 | 0.620904463 | 7.95662749 | 6.66530143 | 6 | 2 |
| [gga-miR-92-3p](http://www.mirbase.org/cgi-bin/query.pl?terms=gga-miR-92-3p) | MIMAT0001109 | 25585.03476 | 13972.95921 | -2.335070754 | 14.34578629 | 1.34788E-07 | 2.38126E-06 | 14.82327639 | 13.59950033 | 703 | 247 |
| [gga-miR-1770](http://www.mirbase.org/cgi-bin/query.pl?terms=gga-miR-1770) | MIMAT0007679 | 109.182225 | 56.570685 | -2.313877266 | 7.496377794 | 0.628703772 | 0.876876313 | 6.96242336 | 5.67944595 | 3 | 1 |
| [gga-miR-183](http://www.mirbase.org/cgi-bin/query.pl?terms=gga-miR-183) | MIMAT0001191 | 109.182225 | 56.570685 | -2.313877266 | 7.496377794 | 0.628703772 | 0.876876313 | 6.96242336 | 5.67944595 | 3 | 1 |
| [gga-miR-365-1-5p](http://www.mirbase.org/cgi-bin/query.pl?terms=gga-miR-365-1-5p) | MIMAT0026630 | 109.182225 | 56.570685 | -2.313877266 | 7.496377794 | 0.628703772 | 0.876876313 | 6.96242336 | 5.67944595 | 3 | 1 |
| [gga-miR-6606-5p](http://www.mirbase.org/cgi-bin/query.pl?terms=gga-miR-6606-5p) | MIMAT0025700 | 109.182225 | 56.570685 | -2.313877266 | 7.496377794 | 0.628703772 | 0.876876313 | 6.96242336 | 5.67944595 | 3 | 1 |
| [gga-miR-99a-5p](http://www.mirbase.org/cgi-bin/query.pl?terms=gga-miR-99a-5p) | MIMAT0001103 | 109.182225 | 56.570685 | -2.313877266 | 7.496377794 | 0.628703772 | 0.876876313 | 6.96242336 | 5.67944595 | 3 | 1 |
| [gga-miR-193a-5p](http://www.mirbase.org/cgi-bin/query.pl?terms=gga-miR-193a-5p) | MIMAT0026787 | 2511.191178 | 1470.837812 | -2.172780712 | 11.0956821 | 0.003887699 | 0.020604804 | 11.47487716 | 10.35256043 | 69 | 26 |
| [gga-miR-15a](http://www.mirbase.org/cgi-bin/query.pl?terms=gga-miR-15a) | MIMAT0001117 | 582.305201 | 339.42411 | -2.166477112 | 9.184665303 | 0.15470229 | 0.390439113 | 9.36803071 | 8.24075662 | 16 | 6 |
| [gga-miR-1682](http://www.mirbase.org/cgi-bin/query.pl?terms=gga-miR-1682) | MIMAT0007569 | 545.911126 | 339.42411 | -2.032224465 | 9.129798588 | 0.211789173 | 0.488035922 | 9.27506686 | 8.24075662 | 15 | 6 |
| [gga-miR-181a-5p](http://www.mirbase.org/cgi-bin/query.pl?terms=gga-miR-181a-5p) | MIMAT0001168 | 363.94075 | 226.28274 | -2.022796951 | 8.659720427 | 0.439814205 | 0.751940414 | 8.6912682 | 7.65817683 | 10 | 4 |
| [gga-miR-206](http://www.mirbase.org/cgi-bin/query.pl?terms=gga-miR-206) | MIMAT0001139 | 3202.678604 | 2093.115348 | -1.948912605 | 11.47939199 | 0.005826044 | 0.029407652 | 11.82567488 | 10.86124599 | 88 | 37 |
| [gga-let-7f-3p](http://www.mirbase.org/cgi-bin/query.pl?terms=gga-let-7f-3p) | MIMAT0026528 | 327.546675 | 226.28274 | -1.823257217 | 8.579309709 | 0.593113131 | 0.876876313 | 8.53965285 | 7.65817683 | 9 | 4 |
| [gga-miR-199-3p](http://www.mirbase.org/cgi-bin/query.pl?terms=gga-miR-199-3p) | MIMAT0003721 | 1128.216326 | 848.560276 | -1.690808524 | 10.12677124 | 0.177052319 | 0.436454553 | 10.32117019 | 9.55982026 | 31 | 15 |
| [gga-miR-194](http://www.mirbase.org/cgi-bin/query.pl?terms=gga-miR-194) | MIMAT0001133 | 436.728901 | 339.42411 | -1.629466527 | 8.950854063 | 0.499674758 | 0.827586317 | 8.9537208 | 8.24075662 | 12 | 6 |
| [gga-miR-203a](http://www.mirbase.org/cgi-bin/query.pl?terms=gga-miR-203a) | MIMAT0001146 | 291.1526 | 226.28274 | -1.623717482 | 8.493971668 | 0.559727541 | 0.876876313 | 8.37021238 | 7.65817683 | 8 | 4 |
| [gga-miR-10b-5p](http://www.mirbase.org/cgi-bin/query.pl?terms=gga-miR-10b-5p) | MIMAT0001148 | 72.78815 | 56.570685 | -1.576342788 | 7.306358021 | 1 | 1 | 6.38323353 | 5.67944595 | 2 | 1 |
| [gga-miR-1781-3p](http://www.mirbase.org/cgi-bin/query.pl?terms=gga-miR-1781-3p) | MIMAT0007693 | 72.78815 | 56.570685 | -1.576342788 | 7.306358021 | 1 | 1 | 6.38323353 | 5.67944595 | 2 | 1 |
| [gga-miR-23b-5p](http://www.mirbase.org/cgi-bin/query.pl?terms=gga-miR-23b-5p) | MIMAT0026546 | 72.78815 | 56.570685 | -1.576342788 | 7.306358021 | 1 | 1 | 6.38323353 | 5.67944595 | 2 | 1 |
| [gga-miR-32-3p](http://www.mirbase.org/cgi-bin/query.pl?terms=gga-miR-32-3p) | MIMAT0026508 | 72.78815 | 56.570685 | -1.576342788 | 7.306358021 | 1 | 1 | 6.38323353 | 5.67944595 | 2 | 1 |
| [gga-let-7c-5p](http://www.mirbase.org/cgi-bin/query.pl?terms=gga-let-7c-5p) | MIMAT0001104 | 91239.94614 | 75691.57663 | -1.537589387 | 16.38195234 | 0.003244942 | 0.018839372 | 16.65760598 | 16.03690072 | 2507 | 1338 |
| [gga-miR-16c-5p](http://www.mirbase.org/cgi-bin/query.pl?terms=gga-miR-16c-5p) | MIMAT0007739 | 473.122976 | 395.994795 | -1.51575108 | 9.077123503 | 0.523804896 | 0.854204908 | 9.06897418 | 8.46246754 | 13 | 7 |
| [gga-miR-200b-5p](http://www.mirbase.org/cgi-bin/query.pl?terms=gga-miR-200b-5p) | MIMAT0026534 | 655.093351 | 565.706851 | -1.471842676 | 9.490748224 | 0.470866639 | 0.792251805 | 9.5377131 | 8.97581321 | 18 | 10 |
| [gga-miR-106-5p](http://www.mirbase.org/cgi-bin/query.pl?terms=gga-miR-106-5p) | MIMAT0001142 | 909.851876 | 791.989591 | -1.46171981 | 9.915079746 | 0.379829855 | 0.734218437 | 10.0111007 | 9.46042112 | 25 | 14 |
| [gga-miR-22-3p](http://www.mirbase.org/cgi-bin/query.pl?terms=gga-miR-22-3p) | MIMAT0007288 | 509.517051 | 452.565481 | -1.430069616 | 9.193295208 | 0.54509069 | 0.875448684 | 9.1756975 | 8.65460128 | 14 | 8 |
| [gga-miR-142-3p](http://www.mirbase.org/cgi-bin/query.pl?terms=gga-miR-142-3p) | MIMAT0001194 | 181.970375 | 169.712055 | -1.354427445 | 8.082780137 | 0.731885362 | 0.957775906 | 7.69475412 | 7.24551811 | 5 | 3 |
| [gga-miR-16-5p](http://www.mirbase.org/cgi-bin/query.pl?terms=gga-miR-16-5p) | MIMAT0001116 | 14157.29519 | 13690.10579 | -1.318986259 | 13.79362067 | 0.095410656 | 0.280931377 | 13.96956202 | 13.56999873 | 389 | 242 |
| [gga-miR-15b-5p](http://www.mirbase.org/cgi-bin/query.pl?terms=gga-miR-15b-5p) | MIMAT0001154 | 291.1526 | 282.853425 | -1.306131483 | 8.584301323 | 1 | 1 | 8.37021238 | 7.97867577 | 8 | 5 |
| [gga-miR-29b-3p](http://www.mirbase.org/cgi-bin/query.pl?terms=gga-miR-29b-3p) | MIMAT0001097 | 291.1526 | 282.853425 | -1.306131483 | 8.584301323 | 1 | 1 | 8.37021238 | 7.97867577 | 8 | 5 |
| [gga-let-7k-5p](http://www.mirbase.org/cgi-bin/query.pl?terms=gga-let-7k-5p) | MIMAT0001182 | 17578.33825 | 18555.1847 | -1.208377134 | 14.15914025 | 0.235435439 | 0.528232263 | 14.28179756 | 14.0086564 | 483 | 328 |
| [gga-miR-21-5p](http://www.mirbase.org/cgi-bin/query.pl?terms=gga-miR-21-5p) | MIMAT0003774 | 4039.74233 | 4355.94275 | -1.18273924 | 12.07651748 | 0.428797623 | 0.751940414 | 12.16057677 | 11.91817622 | 111 | 77 |
| [gga-miR-100-5p](http://www.mirbase.org/cgi-bin/query.pl?terms=gga-miR-100-5p) | MIMAT0001178 | 1091.822251 | 1244.555072 | -1.118453634 | 10.30420515 | 0.803067687 | 1 | 10.27390207 | 10.11175294 | 30 | 22 |
| [gga-miR-1a-3p](http://www.mirbase.org/cgi-bin/query.pl?terms=gga-miR-1a-3p) | MIMAT0001127 | 48768.06056 | 58833.51247 | -1.05736236 | 15.71641896 | 0.709703194 | 0.940356732 | 15.75388914 | 15.67341228 | 1340 | 1040 |
| [gga-miR-20b-5p](http://www.mirbase.org/cgi-bin/query.pl?terms=gga-miR-20b-5p) | MIMAT0001411 | 18633.76642 | 22571.70334 | -1.053042325 | 14.33526601 | 0.75597755 | 0.977239271 | 14.36591407 | 14.29133375 | 512 | 399 |
| [gga-miR-19b-3p](http://www.mirbase.org/cgi-bin/query.pl?terms=gga-miR-19b-3p) | MIMAT0001110 | 4476.47123 | 5543.927137 | -1.029957847 | 12.31316972 | 0.925713664 | 1 | 12.30864466 | 12.26601965 | 123 | 98 |
| [gga-miR-223](http://www.mirbase.org/cgi-bin/query.pl?terms=gga-miR-223) | MIMAT0001140 | 56993.12152 | 71561.91662 | -1.015910774 | 15.96916426 | 0.918144481 | 1 | 15.97873658 | 15.95596123 | 1566 | 1265 |
| [gga-miR-17-5p](http://www.mirbase.org/cgi-bin/query.pl?terms=gga-miR-17-5p) | MIMAT0001114 | 8880.154311 | 11200.99564 | -1.011292569 | 13.3013099 | 0.978766638 | 1 | 13.29672646 | 13.28051848 | 244 | 198 |
| [gga-miR-1306-3p](http://www.mirbase.org/cgi-bin/query.pl?terms=gga-miR-1306-3p) | MIMAT0007329 | 473.122976 | 622.277536 | 1.030759914 | 9.309254699 | 1 | 1 | 9.06897418 | 9.11305622 | 13 | 11 |
| [gga-miR-3538](http://www.mirbase.org/cgi-bin/query.pl?terms=gga-miR-3538) | MIMAT0016389 | 254.758525 | 339.42411 | 1.043619871 | 8.589300029 | 1 | 1 | 8.17819003 | 8.24075662 | 7 | 6 |
| [gga-miR-7](http://www.mirbase.org/cgi-bin/query.pl?terms=gga-miR-7) | MIMAT0001157 | 4622.047531 | 6222.775358 | 1.055381275 | 12.41725935 | 0.825761421 | 1 | 12.35480589 | 12.43263756 | 127 | 110 |
| [gga-miR-103-3p](http://www.mirbase.org/cgi-bin/query.pl?terms=gga-miR-103-3p) | MIMAT0001145 | 545.911126 | 735.418906 | 1.055572093 | 9.502638787 | 1 | 1 | 9.27506686 | 9.35366343 | 15 | 13 |
| [gga-let-7a-5p](http://www.mirbase.org/cgi-bin/query.pl?terms=gga-let-7a-5p) | MIMAT0001101 | 69439.89519 | 94246.76133 | 1.063995538 | 16.31043272 | 0.672705622 | 0.914189691 | 16.26370954 | 16.35320678 | 1908 | 1666 |
| [gga-let-7j-5p](http://www.mirbase.org/cgi-bin/query.pl?terms=gga-let-7j-5p) | MIMAT0001181 | 69439.89519 | 94246.76133 | 1.063995538 | 16.31043272 | 0.672705622 | 0.914189691 | 16.26370954 | 16.35320678 | 1908 | 1666 |
| [gga-let-7f-5p](http://www.mirbase.org/cgi-bin/query.pl?terms=gga-let-7f-5p) | MIMAT0001162 | 36503.25727 | 50630.76314 | 1.087332544 | 15.40026079 | 0.578699733 | 0.876876313 | 15.33598652 | 15.45679281 | 1003 | 895 |
| [gga-miR-146a-5p](http://www.mirbase.org/cgi-bin/query.pl?terms=gga-miR-146a-5p) | MIMAT0001163 | 3129.890454 | 4412.513436 | 1.105033149 | 11.89906292 | 0.685732883 | 0.920097285 | 11.79251726 | 11.93678712 | 86 | 78 |
| [gga-miR-19a-3p](http://www.mirbase.org/cgi-bin/query.pl?terms=gga-miR-19a-3p) | MIMAT0001112 | 946.245951 | 1414.267127 | 1.170785325 | 10.28854728 | 0.61778191 | 0.876876313 | 10.06763045 | 10.29602104 | 26 | 25 |
| [gga-miR-146c-5p](http://www.mirbase.org/cgi-bin/query.pl?terms=gga-miR-146c-5p) | MIMAT0007735 | 23219.41988 | 35300.10748 | 1.191774065 | 14.81926912 | 0.257671028 | 0.557410795 | 14.68331319 | 14.93646509 | 638 | 624 |
| [gga-miR-133c-3p](http://www.mirbase.org/cgi-bin/query.pl?terms=gga-miR-133c-3p) | MIMAT0001176 | 36.394075 | 56.570685 | 1.192167494 | 7.086707396 | 1 | 1 | 5.40041425 | 5.67944595 | 1 | 1 |
| [gga-miR-184-3p](http://www.mirbase.org/cgi-bin/query.pl?terms=gga-miR-184-3p) | MIMAT0001158 | 36.394075 | 56.570685 | 1.192167494 | 7.086707396 | 1 | 1 | 5.40041425 | 5.67944595 | 1 | 1 |
| [gga-miR-26a-5p](http://www.mirbase.org/cgi-bin/query.pl?terms=gga-miR-26a-5p) | MIMAT0001118 | 72.78815 | 113.14137 | 1.2045135 | 7.502304241 | 1 | 1 | 6.38323353 | 6.66530143 | 2 | 2 |
| [gga-miR-140-5p](http://www.mirbase.org/cgi-bin/query.pl?terms=gga-miR-140-5p) | MIMAT0001159 | 145.5763 | 226.28274 | 1.211301134 | 8.088257485 | 1 | 1 | 7.37456583 | 7.65817683 | 4 | 4 |
| [gga-miR-1456-5p](http://www.mirbase.org/cgi-bin/query.pl?terms=gga-miR-1456-5p) | MIMAT0007333 | 181.970375 | 282.853425 | 1.212713088 | 8.311090646 | 1 | 1 | 7.69475412 | 7.97867577 | 5 | 5 |
| [gga-miR-219b](http://www.mirbase.org/cgi-bin/query.pl?terms=gga-miR-219b) | MIMAT0016380 | 254.758525 | 395.994795 | 1.214350034 | 8.674451551 | 0.797511699 | 1 | 8.17819003 | 8.46246754 | 7 | 7 |
| [gga-miR-1b-3p](http://www.mirbase.org/cgi-bin/query.pl?terms=gga-miR-1b-3p) | MIMAT0001175 | 400.334825 | 622.277536 | 1.215860184 | 9.206261521 | 0.840382698 | 1 | 8.82845441 | 9.11305622 | 11 | 11 |
| [gga-let-7i](http://www.mirbase.org/cgi-bin/query.pl?terms=gga-let-7i) | MIMAT0001098 | 63835.20763 | 101940.3745 | 1.251882063 | 16.3145891 | 0.123881589 | 0.345564431 | 16.14229872 | 16.46641609 | 1754 | 1802 |
| [gga-let-7d](http://www.mirbase.org/cgi-bin/query.pl?terms=gga-let-7d) | MIMAT0001161 | 3530.225279 | 5657.068507 | 1.255879822 | 12.16546781 | 0.289999561 | 0.61479907 | 11.96611922 | 12.29516013 | 97 | 100 |
| [gga-miR-199-5p](http://www.mirbase.org/cgi-bin/query.pl?terms=gga-miR-199-5p) | MIMAT0001152 | 218.36445 | 395.994795 | 1.412213469 | 8.594305798 | 0.593113131 | 0.876876313 | 7.95662749 | 8.46246754 | 6 | 7 |
| [gga-miR-7b](http://www.mirbase.org/cgi-bin/query.pl?terms=gga-miR-7b) | MIMAT0001192 | 6514.539433 | 11766.7025 | 1.415655421 | 13.13435146 | 0.051308706 | 0.201434181 | 12.84985696 | 13.35159461 | 179 | 208 |
| [gga-miR-140-3p](http://www.mirbase.org/cgi-bin/query.pl?terms=gga-miR-140-3p) | MIMAT0003722 | 1346.580777 | 2489.110143 | 1.447431675 | 10.92763434 | 0.188770311 | 0.45476484 | 10.57624437 | 11.11110086 | 37 | 44 |
| [gga-miR-6603-3p](http://www.mirbase.org/cgi-bin/query.pl?terms=gga-miR-6603-3p) | MIMAT0025697 | 509.517051 | 961.701646 | 1.475014657 | 9.641945623 | 0.412429952 | 0.740975846 | 9.1756975 | 9.7401676 | 14 | 17 |
| [gga-miR-130c-3p](http://www.mirbase.org/cgi-bin/query.pl?terms=gga-miR-130c-3p) | MIMAT0007734 | 6223.386833 | 12388.98003 | 1.560155445 | 13.15151062 | 0.011514856 | 0.055480671 | 12.78390284 | 13.42593502 | 171 | 219 |
| [gga-miR-30e-3p](http://www.mirbase.org/cgi-bin/query.pl?terms=gga-miR-30e-3p) | MIMAT0026538 | 473.122976 | 961.701646 | 1.587291387 | 9.602021496 | 0.316044516 | 0.638934768 | 9.06897418 | 9.7401676 | 13 | 17 |
| [gga-miR-130a-3p](http://www.mirbase.org/cgi-bin/query.pl?terms=gga-miR-130a-3p) | MIMAT0001167 | 1528.551152 | 3111.387679 | 1.593785196 | 11.18216299 | 0.081098099 | 0.260496925 | 10.75899593 | 11.4328985 | 42 | 55 |
| [gga-miR-301a-3p](http://www.mirbase.org/cgi-bin/query.pl?terms=gga-miR-301a-3p) | MIMAT0001166 | 109.182225 | 226.28274 | 1.597396094 | 7.965289618 | 1 | 1 | 6.96242336 | 7.65817683 | 3 | 4 |
| [gga-let-7j-3p](http://www.mirbase.org/cgi-bin/query.pl?terms=gga-let-7j-3p) | MIMAT0026542 | 218.36445 | 452.565481 | 1.610762017 | 8.679374769 | 0.439814205 | 0.751940414 | 7.95662749 | 8.65460128 | 6 | 8 |
| [gga-miR-130b-3p](http://www.mirbase.org/cgi-bin/query.pl?terms=gga-miR-130b-3p) | MIMAT0001165 | 10153.94694 | 21723.14307 | 1.676817568 | 13.91686941 | 0.001553373 | 0.010291094 | 13.49009245 | 14.23605413 | 279 | 384 |
| [gga-miR-146b-5p](http://www.mirbase.org/cgi-bin/query.pl?terms=gga-miR-146b-5p) | MIMAT0003351 | 509.517051 | 1131.413701 | 1.733597324 | 9.766181919 | 0.211409834 | 0.488035922 | 9.1756975 | 9.97437979 | 14 | 20 |
| [gga-miR-23b-3p](http://www.mirbase.org/cgi-bin/query.pl?terms=gga-miR-23b-3p) | MIMAT0001186 | 16158.96932 | 36148.66776 | 1.753497844 | 14.62520874 | 0.000305135 | 0.002310311 | 14.16034041 | 14.97073391 | 444 | 639 |
| [gga-miR-15b-3p](http://www.mirbase.org/cgi-bin/query.pl?terms=gga-miR-15b-3p) | MIMAT0026524 | 72.78815 | 169.712055 | 1.774647215 | 7.675401303 | 1 | 1 | 6.38323353 | 7.24551811 | 2 | 3 |
| [gga-miR-181b-5p](http://www.mirbase.org/cgi-bin/query.pl?terms=gga-miR-181b-5p) | MIMAT0001151 | 72.78815 | 169.712055 | 1.774647215 | 7.675401303 | 1 | 1 | 6.38323353 | 7.24551811 | 2 | 3 |
| [gga-miR-99a-3p](http://www.mirbase.org/cgi-bin/query.pl?terms=gga-miR-99a-3p) | MIMAT0006781 | 218.36445 | 509.136166 | 1.809310564 | 8.759897316 | 0.319467384 | 0.638934768 | 7.95662749 | 8.82412846 | 6 | 9 |
| [gga-miR-456-3p](http://www.mirbase.org/cgi-bin/query.pl?terms=gga-miR-456-3p) | MIMAT0003777 | 3311.860829 | 7919.89591 | 1.873381786 | 12.41470648 | 0.001521352 | 0.010291094 | 11.87402479 | 12.78050494 | 91 | 140 |
| [gga-miR-1451-5p](http://www.mirbase.org/cgi-bin/query.pl?terms=gga-miR-1451-5p) | MIMAT0007324 | 145.5763 | 395.994795 | 2.094885105 | 8.418623986 | 0.401742164 | 0.734218437 | 7.37456583 | 8.46246754 | 4 | 7 |
| [gga-miR-16-2-3p](http://www.mirbase.org/cgi-bin/query.pl?terms=gga-miR-16-2-3p) | MIMAT0031058 | 145.5763 | 395.994795 | 2.094885105 | 8.418623986 | 0.401742164 | 0.734218437 | 7.37456583 | 8.46246754 | 4 | 7 |
| [gga-miR-2131-3p](http://www.mirbase.org/cgi-bin/query.pl?terms=gga-miR-2131-3p) | MIMAT0026920 | 145.5763 | 395.994795 | 2.094885105 | 8.418623986 | 0.401742164 | 0.734218437 | 7.37456583 | 8.46246754 | 4 | 7 |
| [gga-miR-126-3p](http://www.mirbase.org/cgi-bin/query.pl?terms=gga-miR-126-3p) | MIMAT0001169 | 291.1526 | 791.989591 | 2.113360868 | 9.219252782 | 0.15470229 | 0.390439113 | 8.37021238 | 9.46042112 | 8 | 14 |
| [gga-miR-196-5p](http://www.mirbase.org/cgi-bin/query.pl?terms=gga-miR-196-5p) | MIMAT0001121 | 363.94075 | 1018.272331 | 2.177233548 | 9.522481053 | 0.080304383 | 0.260496925 | 8.6912682 | 9.82253604 | 10 | 18 |
| [gga-let-7g-5p](http://www.mirbase.org/cgi-bin/query.pl?terms=gga-let-7g-5p) | MIMAT0001160 | 5313.534957 | 15443.79702 | 2.277329189 | 13.27700016 | 2.83854E-06 | 3.76106E-05 | 12.55590991 | 13.74387917 | 146 | 273 |
| [gga-miR-214](http://www.mirbase.org/cgi-bin/query.pl?terms=gga-miR-214) | MIMAT0007751 | 1346.580777 | 4242.80138 | 2.464608048 | 11.40377136 | 0.000269375 | 0.002196445 | 10.57624437 | 11.88021831 | 37 | 75 |
| [gga-miR-2954](http://www.mirbase.org/cgi-bin/query.pl?terms=gga-miR-2954) | MIMAT0014448 | 1819.703752 | 6053.063303 | 2.603304121 | 11.88314071 | 1.41198E-05 | 0.00014967 | 11.01040148 | 12.39275215 | 50 | 107 |
| [gga-miR-107-3p](http://www.mirbase.org/cgi-bin/query.pl?terms=gga-miR-107-3p) | MIMAT0001147 | 181.970375 | 622.277536 | 2.635894284 | 8.841105129 | 0.08848945 | 0.26799662 | 7.69475412 | 9.11305622 | 5 | 11 |
| [gga-miR-215-5p](http://www.mirbase.org/cgi-bin/query.pl?terms=gga-miR-215-5p) | MIMAT0001134 | 181.970375 | 622.277536 | 2.635894284 | 8.841105129 | 0.08848945 | 0.26799662 | 7.69475412 | 9.11305622 | 5 | 11 |
| [gga-miR-1306-5p](http://www.mirbase.org/cgi-bin/query.pl?terms=gga-miR-1306-5p) | MIMAT0026763 | 109.182225 | 395.994795 | 2.762617148 | 8.321672743 | 0.239199515 | 0.528232263 | 6.96242336 | 8.46246754 | 3 | 7 |
| [gga-miR-142-5p](http://www.mirbase.org/cgi-bin/query.pl?terms=gga-miR-142-5p) | MIMAT0001193 | 2511.191178 | 9503.875092 | 2.963009499 | 12.47547275 | 5.34527E-08 | 1.1332E-06 | 11.47487716 | 13.04350517 | 69 | 168 |
| [gga-miR-101-3p](http://www.mirbase.org/cgi-bin/query.pl?terms=gga-miR-101-3p) | MIMAT0001185 | 181.970375 | 735.418906 | 3.110288015 | 8.983098512 | 0.03847433 | 0.163131159 | 7.69475412 | 9.35366343 | 5 | 13 |
| [gga-miR-27b-3p](http://www.mirbase.org/cgi-bin/query.pl?terms=gga-miR-27b-3p) | MIMAT0001187 | 36.394075 | 226.28274 | 4.406463868 | 7.681231163 | 0.381172953 | 0.734218437 | 5.40041425 | 7.65817683 | 1 | 4 |
| [gga-miR-24-3p](http://www.mirbase.org/cgi-bin/query.pl?terms=gga-miR-24-3p) | MIMAT0001188 | 1856.097827 | 13180.96962 | 5.554859201 | 12.76066932 | 5.24643E-17 | 3.69876E-15 | 11.03895692 | 13.51532622 | 51 | 233 |
| [gga-miR-126-5p](http://www.mirbase.org/cgi-bin/query.pl?terms=gga-miR-126-5p) | MIMAT0003723 | 218.36445 | 1640.549867 | 5.780281516 | 9.834352235 | 5.25415E-05 | 0.000464117 | 7.95662749 | 10.50998755 | 6 | 29 |
| [gga-miR-30c-1-3p](http://www.mirbase.org/cgi-bin/query.pl?terms=gga-miR-30c-1-3p) | MIMAT0031105 | 109.182225 | 1244.555072 | 8.588722419 | 9.403611068 | 2.95077E-05 | 0.000284347 | 6.96242336 | 10.11175294 | 3 | 22 |
| [gga-miR-222a](http://www.mirbase.org/cgi-bin/query.pl?terms=gga-miR-222a) | MIMAT0001107 | 36.394075 | 452.565481 | 8.692192367 | 8.222853694 | 0.024634949 | 0.108804359 | 5.40041425 | 8.65460128 | 1 | 8 |

RC**,** Resistant control; RI, Resistant infection; RPM, reads per million; fc, fold change; CPM, counts per million; bh.pval, Benjamini-Hochberg adjusted P value
