# Supplementary material for: Exosomal miRNA profiling from H5N1 avian influenza virus-infected chickens
Source: Vet Res. 2021 Mar 3;52:36. doi: 10.1186/s13567-021-00892-3 (PMC7931527; doi:10.1186/s13567-021-00892-3)
Supplement: Supplementary file 8 — Additional file 8. Gene ontology analysis. (A) Biological process (B) Cellular component (C) Molecular function. Target categorized in specific functional groups according to gene ontology using Fisher’s exact test (p < 0.01). [file 13567_2021_892_MOESM8_ESM.docx]

**Figure S5.** Gene ontology analysis. (A) Biological process (B) Cellular component (C) Molecular function. Target categorized in specific functional groups according to gene ontology using Fisher's exact test (*p*<0.01).
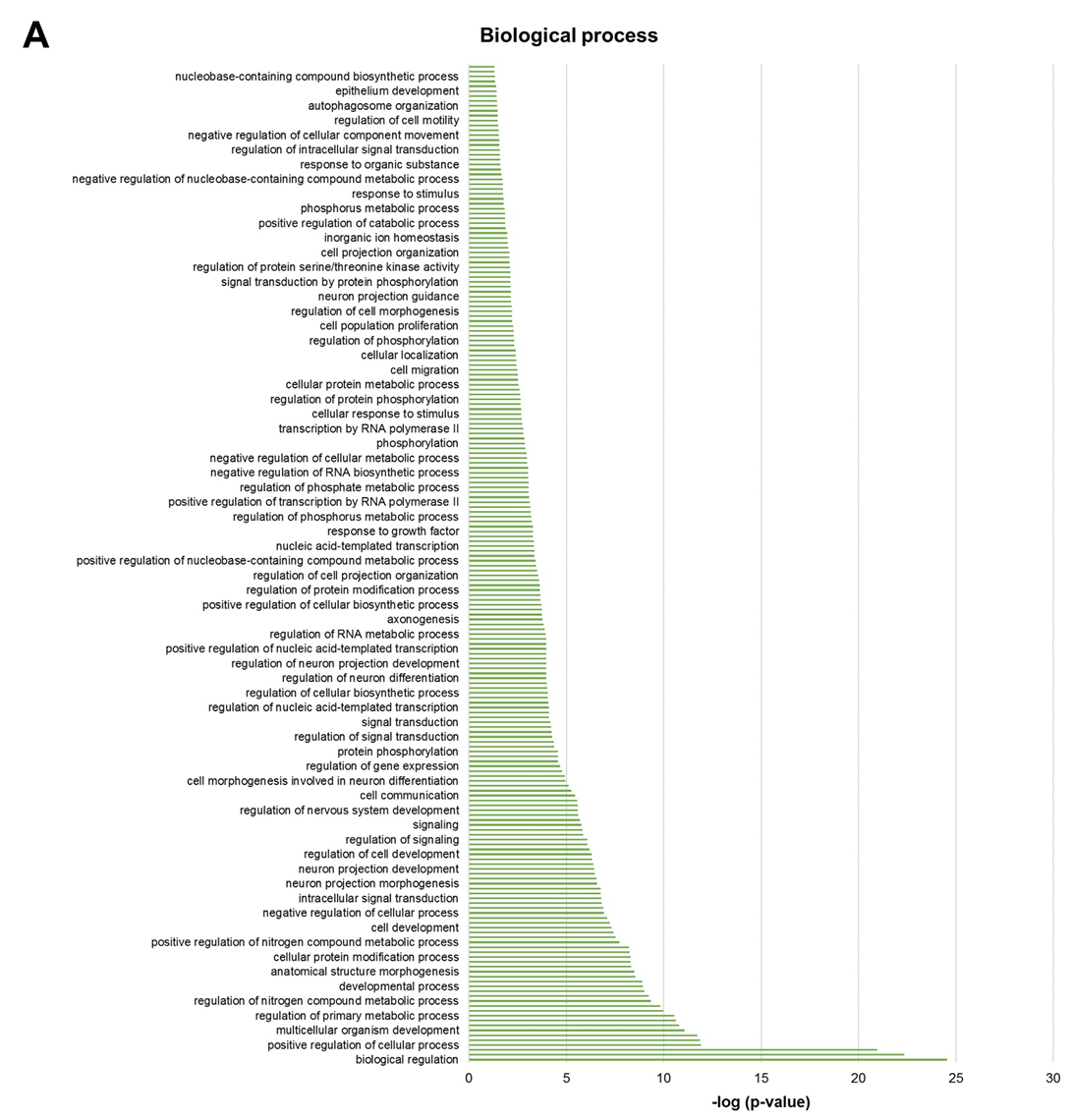


**
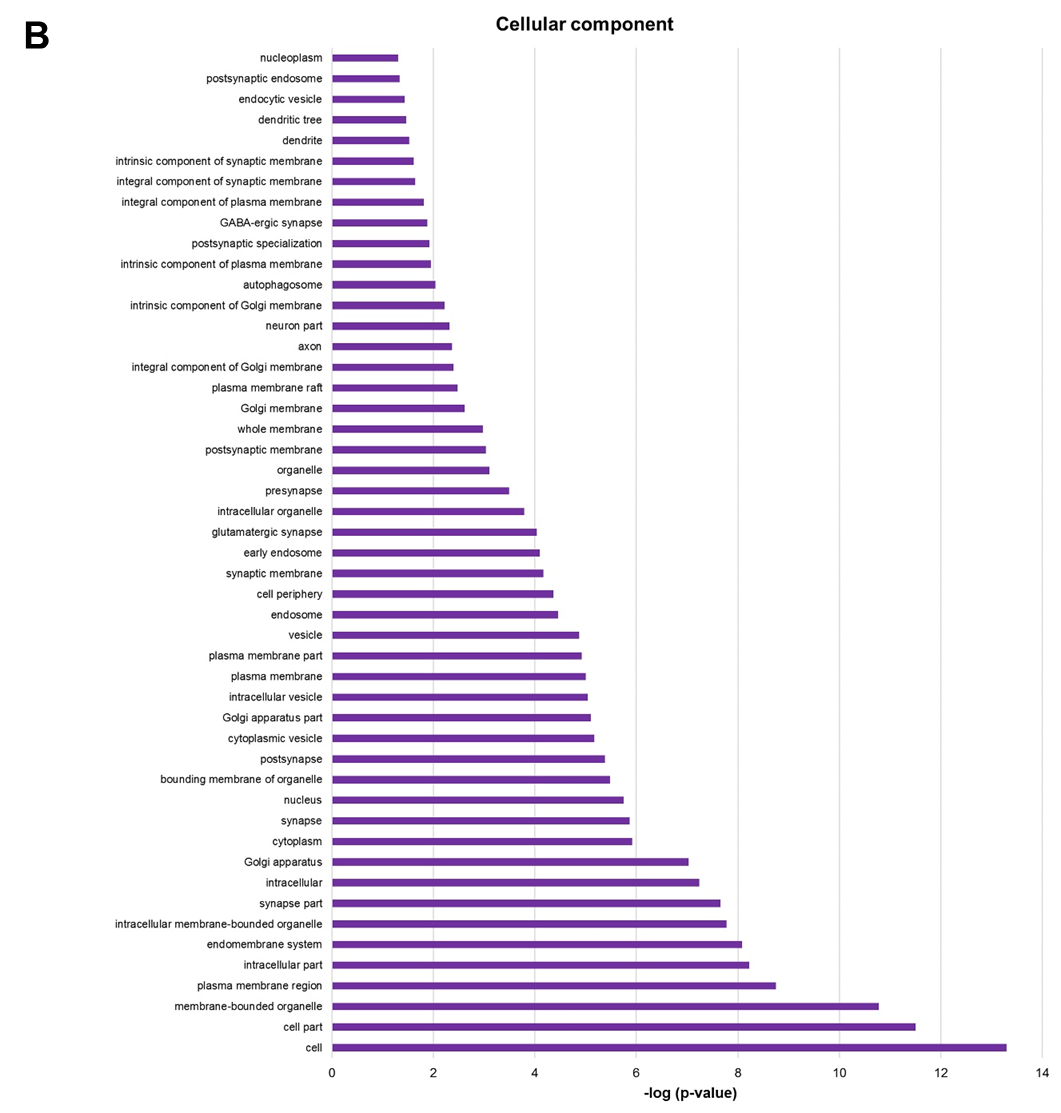
**

**
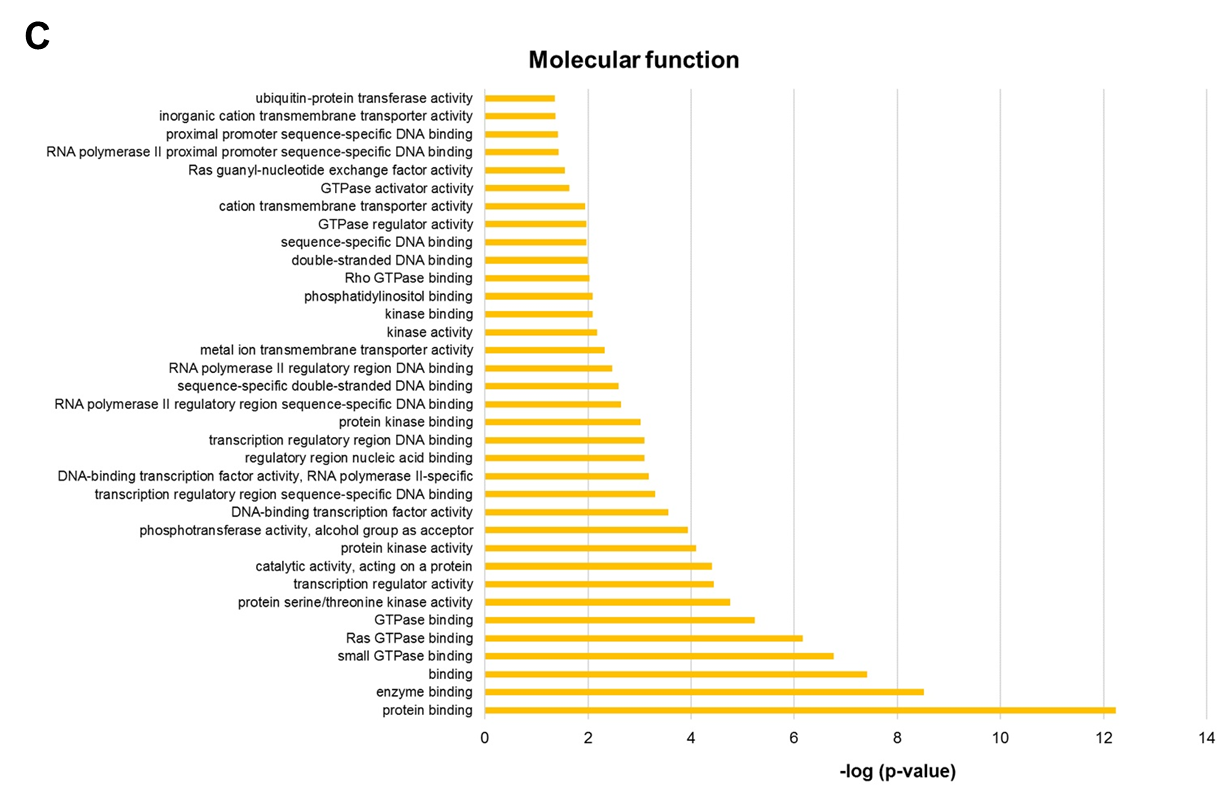
**
